# Temporal switching of an amphiphilic self-assembly by a chemical fuel-driven conformational response†
†Electronic supplementary information (ESI) available: Experimental section, synthetic procedures and supporting figures. See DOI: 10.1039/c7sc01730h



**DOI:** 10.1039/c7sc01730h

**Published:** 2017-07-11

**Authors:** Krishnendu Jalani, Shikha Dhiman, Ankit Jain, Subi J. George

**Affiliations:** a Supramolecular Chemistry Laboratory , New Chemistry Unit , Jawaharlal Nehru Centre for Advanced Scientific Research (JNCASR) , Jakkur , Bangalore , India-560064 . Email: ajdendros@gmail.com ; Email: george@jncasr.ac.in ; http://www.jncasr.ac.in/george

## Abstract

The spatial and temporal control of self-assemblies is the latest scientific hurdle in supramolecular chemistry which is inspired by the functioning of biological systems fueled by chemical signals. In this study, we work towards alleviating this scenario by employing a unique amphiphilic foldamer that operates under the effect of a chemical fuel. The conformational changes in the foldamer amplify into observable morphological changes in its amphiphilic assembly that are controlled by external molecular cues (fuel). We take advantage of this redox responsive foldamer to affect its conformation in a temporal manner by an enzymatic pathway. The temporal characteristics of the transient conformation/assembly can be modulated by varying the concentrations of the fuel and enzyme. We believe that such a design strategy can have positive consequences in designing molecular and supramolecular systems for future active, adaptive and autonomous materials.

## Abstract 2

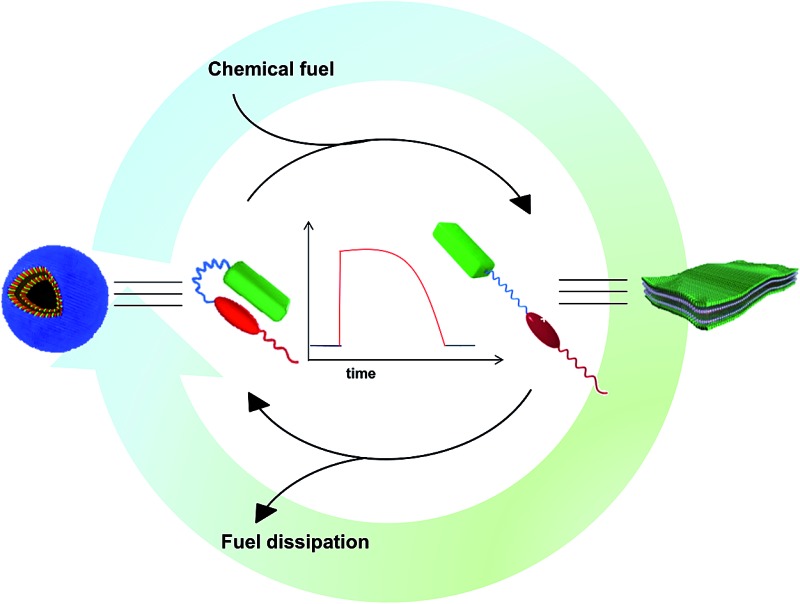
A unique redox active amphiphilic foldamer that undergoes transient conformation changes that amplify into observable morphology changes in its self-assembly.

## Introduction

In the past few decades, the synthesis of supramolecular materials has seen various changes that have targeted their spatial design and precision.^1^ These equilibrium assemblies, however, are restricted to spatial control and are temporally passive. Recently, supramolecular chemistry has started evolving around the time domain to target next generation materials which are inspired by biological out-of-equilibrium systems so that they would have active, adaptive and autonomous behaviour.^2^ Although light responsive synthetic assemblies with active control^3^ have been investigated, natural systems that function out-of-equilibrium, such as circadian rhythms, ion pumps and microtubules, utilize chemical signals for their autonomous and temporal control. Nature also exploits the amphiphilic nature of molecules for a variety of functions, among which are transmembrane proteins, which because of their amphiphilic nature can undergo spatial organization into the hydrophobic lipid bilayer and translocate hydrophilic substances when triggered by a chemical fuel. Among these is transmembrane protein complex coenzyme Q-cytochrome c reductase^4^ (also known as cytochrome bc_1_ complex) which undergoes a transient reduction induced conformational change for the functioning of an electron transport chain when triggered by a chemical fuel. A step towards the next technological hurdle for complex matter can be taken by extending this natural scenario to synthetic supramolecular systems to temporally control their molecular organization and resultant morphology.

Recently, chemical fuel-driven strategies for temporal regulation have been explored in supramolecular systems.^5^ Although these systems target transient self-assembly,^6^ taking inspiration from non-equilibrium polymerization of microtubules and other non-equilibrium systems such as the conformational switching^7^ of transmembrane proteins remains a challenge to be explored. A very recent example from our group depicted the transient conformational switching of a supramolecular assembly^7*a*^ and in an interesting study by Walther’s group temporal control over pH was utilised to transiently control conformations in i-motif DNA.^7*b*^ In this context, we envisage that a bio-inspired feasible route for temporal manifestation would be able to switch the conformation of an amphiphilic supramolecular motif to change its hydrophilic to hydrophobic ratio through a chemical fuel-driven process that displays the importance of conformational switching in a unique way. This chemical signal can then be connected *via* networks to stimulate changes in the supramolecular system, giving the material multi-parameter temporal control.

**Scheme 1:**
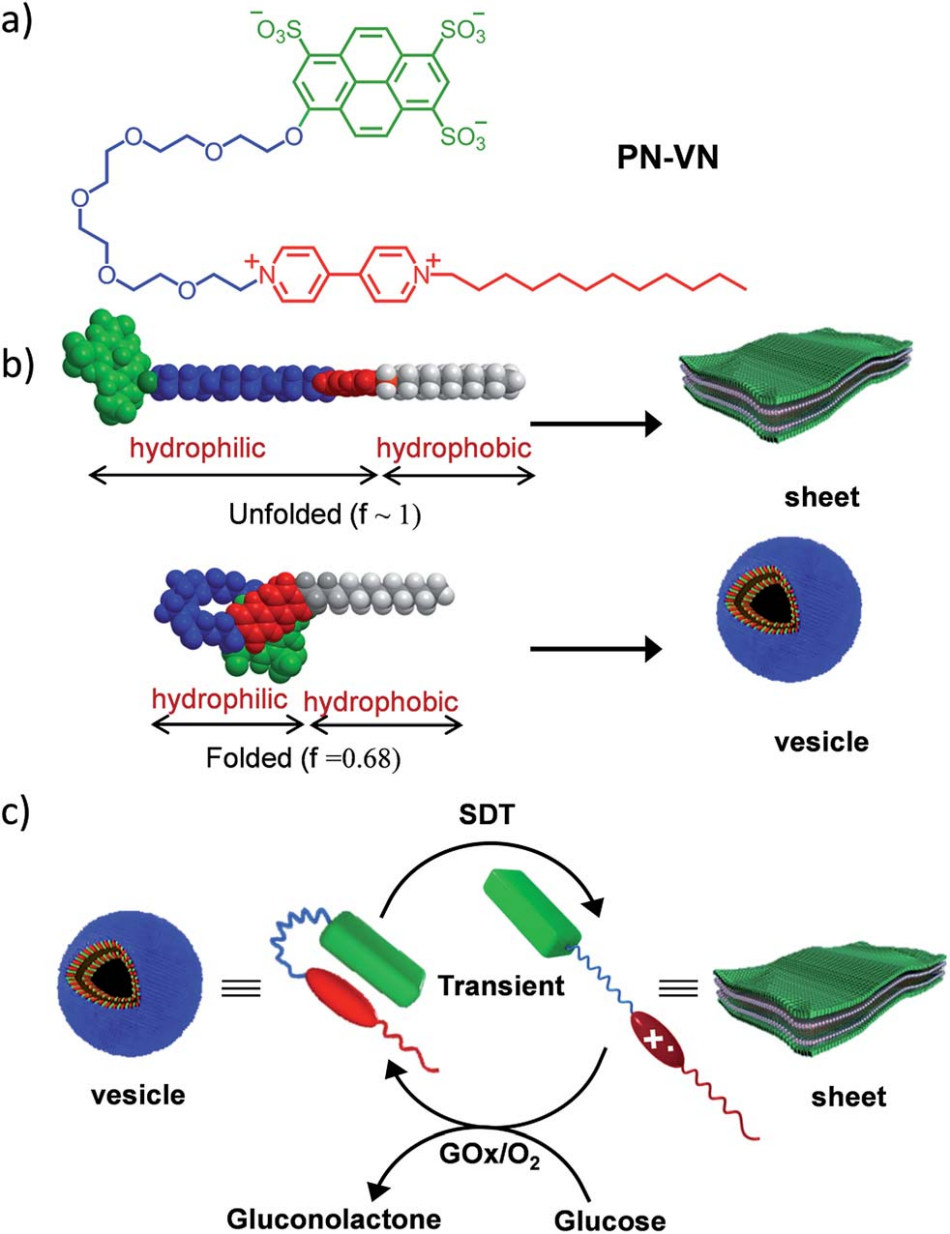
Design of the amphiphilic foldamer. (a) Molecular structure of the **PN–VN** amphiphilic foldamer. (b) Molecular models of the folded and unfolded conformations of **PN–VN**, which have significant differences in packing factors, and a schematic representation of the corresponding amphiphilic self-assembly. (c) Schematic representation for the transient conformational response of the foldamer driven by a chemical fuel and mediated *via* an enzymatic pathway. **PN–VN** = pyranine–viologen foldamer, **SDT** = sodium dithionite, GO_*x*_ = glucose oxidase.

Herein we introduce a charge transfer^8^ (CT) interaction governed amphiphilic foldamer^9^ that exhibits temporal control over its switchable conformation and self-assembly by a bio-inspired chemical fuel-driven strategy. Our unique molecular design of the amphiphilic foldamer, **PN–VN**, consists of an electron donor (pyranine) connected through a flexible hydrophilic hexaethylene glycol connector to an electron acceptor (viologen), which is further attached to a hydrophobic tail endowing its amphiphilic nature (Scheme 1a). The hydrophilic connector acts as the crucial part of the design and is responsible for joining the two functional moieties pyranine and viologen that form the CT complex, which governs the folding of **PN–VN**, hence, a stimuli induced disruption of the CT interaction should reflect in the unfolding of the molecule. So, this CT interaction imparts the fabled stimuli responsiveness for the conformational control of a foldamer. Since this novel molecule can exist in folded and unfolded conformational states, there is a change in its packing factor (*f*) from ∼0.68 to 1 which results in its unique spatial morphological manifestation (Scheme 1b).

In the present study, we show the temporal control between the folded and unfolded conformations driven by a chemical fuel (Scheme 1c). Since we hypothesized that the foldamer should show different morphological structures in its two conformations, folded and unfolded, we first investigated the two conformations using spectroscopic techniques and the morphologies by imaging with TEM, AFM and confocal microscopy.

In order to thoroughly investigate the unfolded conformation it needed to be stable, hence we utilized a passive stimulus such as a host–guest interaction between cucurbit[7]uril (CB[7]) and viologen to characterize this state. Then, we used the redox active capability of viologen to control the conformational transition in temporally active regimes where sodium dithionite (**SDT**) acted as the chemical fuel to drive the change. The decaying pathway takes advantage of dissolved oxygen as well as enzymatic assistance using glucose oxidase (GO_*x*_).

## Results and discussion

**Fig. 1:**
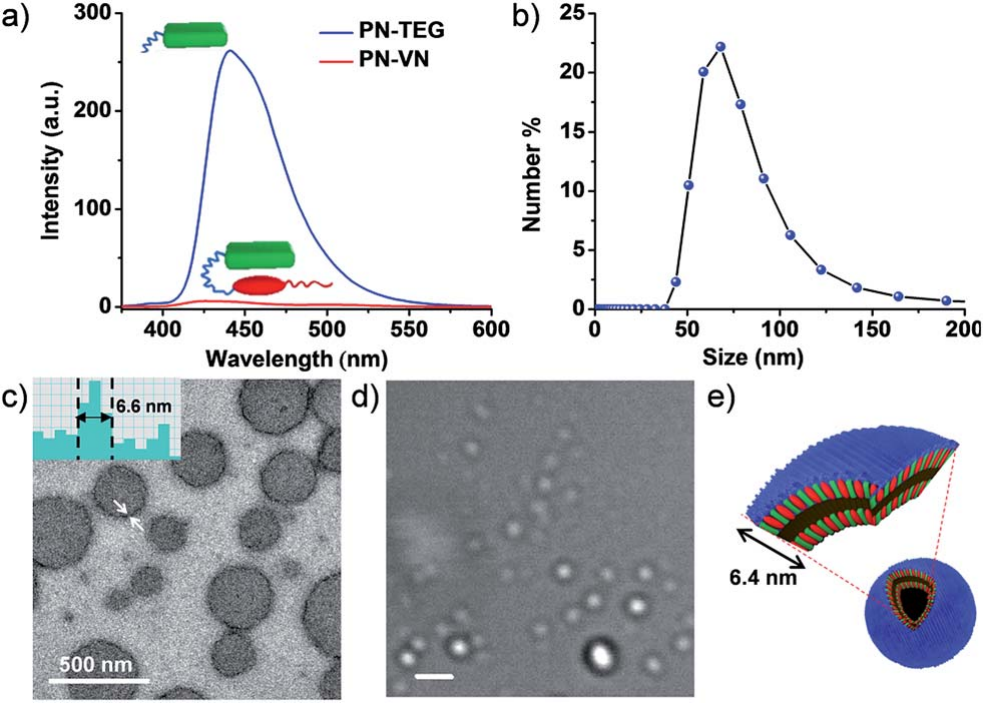
Folded conformation of **PN–VN** and its vesicular self-assembly. (a) Comparison of the quenched emission of **PN–VN** along with the emission of the model molecule **PN-TEG** (*λ*_exc_ = 320 nm). (b) Dynamic Light Scattering (DLS) data of **PN–VN** vesicle assembly. (c) TEM and (d) bright field confocal microscopy images (scale bar is 2 μm). (e) Schematic representation of the **PN–VN** vesicles. The inset of (c) is a TEM histogram showing a vesicle wall thickness of 6.6 nm which matches closely with the bilayer distance of 6.4 nm of the amphiphilic foldamer. [**PN–VN**] = 10^–4^ M, [**PN-TEG**] = 10^–4^ M, H_2_O, 25 °C.

**PN–VN** was synthesized and appropriately characterized (Schemes S1–S3†). First we investigated the self-assembly characteristics of this novel amphiphilic foldamer **PN–VN** in its folded and unfolded conformations. **PN–VN** in water exhibited a red-shifted absorption band at 480 nm and quenched emission compared to the glycol-tethered pyranine control derivative (**PN-TEG**), characteristic of pyranine–viologen CT interactions (Fig. 1a and S1†).^10^ Concentration dependent studies on **PN–VN** showed a linear decrease in the CT absorbance at 480 nm with dilution, suggesting an intramolecular CT driven folded native conformation for **PN–VN** in water (Fig. S2†).

Dynamic light scattering (DLS), TEM and cryo transmission electron microscopy (TEM) studies revealed the self-assembly of **PN–VN** into vesicles with an average size of 70 nm (Fig. 1b and c and S3†). We have also plotted the histogram for the size distribution of the vesicles inspected in the cryo-TEM images which reiterates the same observation (Fig. S4†). The confocal microscopy images further support the formation of vesicles, however, due to low resolution, smaller vesicles could not be captured (Fig. 1c–e). The packing factor,^11^ manifested by the folded conformation and its amphiphilic character, clearly justifies the vesicular morphology (Scheme S4†). Detailed TEM analysis of the vesicles revealed a wall thickness of 6.6 nm, which is twice the calculated length of the folded **PN–VN** conformation, thus confirming a bilayer packing as shown in Fig. 1c.

**Fig. 2:**
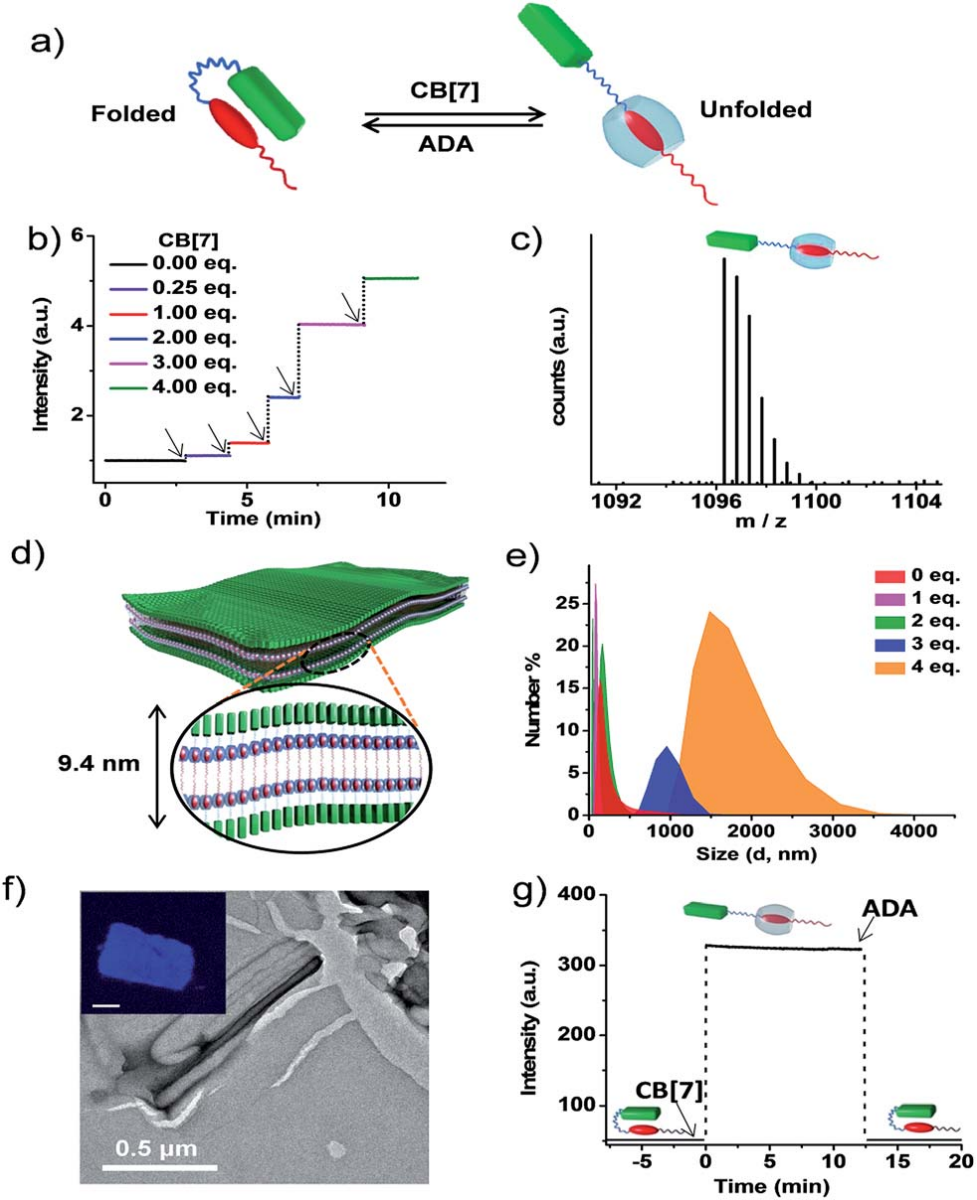
Unfolded conformation of **PN–VN** and its two-dimensional self-assembly into sheets. (a) Schematic representation of the reversible switching of **PN–VN** between its folded and unfolded conformation driven by CB[7] and ADA. (b) Plot of emission kinetics upon the sequential addition of CB[7], represented by the black arrows, monitored at 430 nm showing fast emission increases. (c) HR-MS showing the 1 : 1 complex formation of the foldamer with CB[7]. The experiment was performed with 4 eq. of CB[7] and 1 eq. of **PN–VN**. (d) Schematic representation of the sheet morphology with lamellar packing showing a bilayer distance of 9.4 nm. (e) Change in DLS size for the vesicle to sheet transformation with the addition of CB[7] to **PN–VN**. (f) TEM image of the sheet (**PN–VN** + 4 eq. CB[7]), the inset shows the confocal fluorescence microscopy image of the sheet (scale bar: 2 μm). (g) Emission changes for the passive folding–unfolding cycle by the sequential addition of CB[7] (4 eq.) and ADA (4 eq.) to **PN–VN**. [**PN–VN**] = 10^–4^ M, H_2_O, 25 °C, CB[7] = cucurbit[7]uril, ADA = adamantylamine.

The association constant (*K*_a_) of the intramolecular CT between pyranine and viologen in the folded state was calculated to be of the order of 10^4^–10^5^ (Fig. S5 and S6†).^12^ In order to characterize the morphological state of the unfolded conformation of **PN–VN**, we have exploited the well-established host–guest chemistry of viologen and cucurbiturils.^13^ An approximate value of *K*_a_ (∼ 10^4^–10^5^) clearly suggests that CB[7], because of its higher association constant (10^5^ M^–1^ to 10^6^ M^–1^) with viologen reported in the literature, would overcome the intramolecular CT interaction and should be capable of unfolding the **PN–VN** foldamer (Fig. 2a). In the present system, the unfolding process can be easily probed by the emission increase of pyranine from its CT quenched state. Interestingly, the stepwise titration of CB[7] with the **PN–VN** foldamer resulted in an instantaneous increase in pyranine emission, suggesting that a very fast unfolding process occurs, which is finally saturated with 4 equivalents of CB[7] (Fig. 2b and S7†). Although viologen and CB[7] form a 1 : 1 complex, 4 equivalents of CB[7] is perhaps required as a consequence of only one site access *via* a long alkyl chain which restricts the threading of CB[7] and the subsequent binding to the viologen.^14^ Furthermore, the host–guest complexation and the unfolding process is evident from high-resolution mass-spectrometry (HR-MS) measurements of a 1 : 4 **PN–VN** : CB[7] solution which showed a peak at *m*/*z* = 1096.3251 corresponding to [**PN–VN** + CB[7] + 3H]^2+^ (Fig. 2c). The unfolding process is further corroborated by the disappearance of the CT absorption band at 480 nm (Fig. S7c†).

We further anticipate that the unfolding of this amphiphilic foldamer would result in a change in its assembly, due to the significant difference in the packing factor (*f*) of the folded conformation (*f* = 0.68) with respect to the unfolded conformation (*f* ∼ 1) (Schemes S4 and S5†). The number average size distribution data obtained from the detailed DLS probing during the titration of CB[7] with **PN–VN** indeed show the appearance of larger assemblies with the gradual disappearance of the smaller vesicles (Fig. 2e). We have also plotted the data as intensity distributions which follow the same trend (Fig. S8†). Furthermore, the TEM studies revealed a transformation of the vesicles to two-dimensional (2D) sheet-like nanostructures which is consistent with the change in the packing factor of the unfolded amphiphile (Fig. 2f and S9†). The AFM height analysis of the sheet showed 9.6 nm as the minimum height of the structures (Fig. S10†). This relates well with the calculated length of a bilayer consisting of unfolded **PN–VN** molecules, suggesting that bilayers are organized along the thickness of the sheet (Fig. 2d). Furthermore, the confocal microscopy image of the sheet exhibited pyranine fluorescence, validating the presence of unfolded pyranine groups on its surface and the absence of CT interactions (inset, Fig. 2f and S11†). As additional evidence, the zeta-potential measurement for the sheets clearly confirms the existence of a negatively charged sheet surface (Fig. S12†).

To get further insight into the vesicle to sheet transformation we have determined the critical aggregation concentration (CAC) of the vesicles and the sheets from the DLS studies and also from the percentage of unfolded **PN–VN** monomers during the titration with CB[7] which was quantified from the corresponding emission changes (Fig. S13 and S14†). The dilution dependent DLS studies of the **PN–VN** sheets (unfolded monomers) with 4 eq. of CB[7] and the vesicles (folded monomers) showed CACs of 3.4 × 10^–5^ M and 5 × 10^–5^ M, respectively. As a result, at the initial stages of the titration (∼1 equivalent of CB[7], which is below the CAC of the sheets) only vesicles and unfolded monomers are present. On the other hand, in a regime where the concentration of the unfolded monomers is above the CAC of the sheets (∼2 equivalents of CB[7]), both vesicles and sheets co-exist which is evident from the bimodal distribution in the DLS studies and from the TEM analysis (Fig. S15†). Upon further increasing the amount of CB[7], the concentration of the folded monomers goes below the CAC of the vesicles, resulting in the presence of only sheets in solution, which is also marked by a sudden increase in the emission resulting from the unfolded monomers.

Next, to verify the reversibility of the system to reform vesicles, adamantylamine (ADA) was used. ADA has a higher association constant (*K*_a_ = 10^12^ M^–1^) with CB[7] and thus it expels viologen from the CB[7] pocket leading to the formation of folded **PN–VN**.^15^ Thus, the addition of 4 equivalents of ADA to the **PN–VN** + CB[7] mixture resulted in the morphological switching from sheet to vesicle which is consistent with the conventional passive assembly (Fig. 2g and S16†).^16^

With the understanding of the effect of the conformational switching of the amphiphilic foldamer on the morphology by a passive stimuli–responsive transition, we then envisaged the final target of achieving temporal switching of the two self-assembled nanostructures *via* a chemical fuel-driven conformational response. Inspired by the redox responsive conformational change in coenzyme Q-cytochrome c reductase, the well-known redox chemistry of viologen was targeted. Since CT is responsible for the native folded conformational state of **PN–VN**, the weakening of this CT interaction should reflect in the unfolding of the foldamer.

**Fig. 3:**
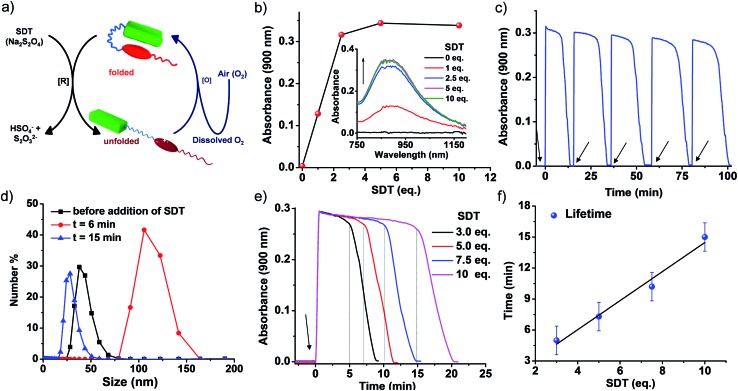
Transient conformational switching *via* a non-enzymatic decay route. (a) Schematic representation of transient conformational switching between the unfolding of **PN–VN** by **SDT** as the reducing agent and the non-enzymatic re-folding by dissolved oxygen. (b) The appearance of the viologen radical cation (VN˙^+^) assembly band upon reduction with **SDT**, with the inset showing the corresponding absorption spectra. (c) The absorption changes at *λ* = 900 nm (characteristic of VN˙^+^ assembly) depicting the transient conformational switching in the presence of **SDT** and O_2_ and the refuelling of the system by subsequent addition of **SDT**, [**SDT**] = 5 eq. (d) Dynamic light scattering data depicting the change in size over the transient cycle, [**SDT**] = 5 eq. (e) Absorption changes at *λ* = 900 nm depicting the transient conformational switching in the presence of **SDT** and O_2_ and modulation of the lifetime by varying the equivalents of **SDT**, [**SDT**] = 3–10 eq. (f) The lifetime of transient conformation by variation of **SDT** showing a linear increase. The data plotted are the average of four different experiments with their standard deviations. [**PN–VN**] = 10^–4^ M.

To realize this, **PN–VN** was treated with a chemical fuel, sodium dithionite (**SDT**), a known reducing agent, to reduce the viologen dication (VN^2+^) to the radical cation (VN˙^+^) resulting in the decreased acceptor strength of viologen. This led to a weakening of the CT interaction and the subsequent unfolding of the foldamer which was verified by the appearance of a new band at 900 nm corresponding to VN˙^+^ in its assembled state (Fig. 3b and S17†).^6g,17^ As we believed, the redox stimuli induced unfolding of **PN–VN** also leads to a morphological transition from vesicles to sheets as visualized by TEM (Fig. S18a†), verifying that sheet formation results from the unfolding of **PN–VN** and is independent of CB[7]. This is further justified by the packing factor of unfolded **PN–VN˙^+^**, which remains unchanged with and without CB[7] because the calculation of the packing factor involves only the interfacial area of the hydrophilic part (Scheme S5†).^18^

**Fig. 4:**
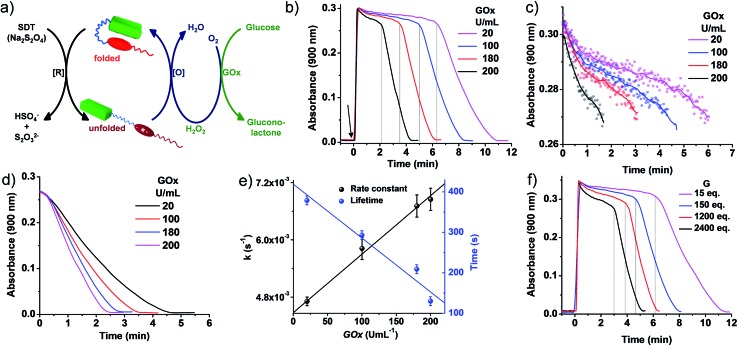
Transient conformational switching *via* an enzymatic decay route. (a) Schematic representation of transient conformational switching between the unfolding of **PN–VN** by **SDT** as the reducing agent and the enzymatic re-folding by Glucose Oxidase (GO_*x*_) in the presence of glucose and dissolved oxygen. (b)–(d) Absorption changes at *λ* = 900 nm (*A*_900_) depicting transient conformational switching *via* enzymatic oxidation. (c)–(e) Modulation of the decay rate and lifetime of transient conformation by variation of GO_*x*_ units, [**SDT**] = 5 eq., [glucose] = 100 eq., GO_*x*_ = 20–200 U mL^–1^. (b) Enzymatic oxidation due to *in situ* synthesized H_2_O_2_ by GO_*x*_ in the presence of glucose and modulation of the lifetime of the transient conformational state, [**SDT**] = 5 eq., [glucose] = 100 eq., GO_*x*_ = 20–200 U mL^–1^. Graphs extracted from Fig. 1(b) by cropping the decay region from *t* = 0 to (c) *A*_900_ > 0.27 and (d) *A*_900_ < 0.27 where the time has been subtracted from the respective time where *A*_900_ = 0.27. The data plotted in (e) are the average of two different experiments with their standard deviations. (f) Effect of glucose on the rate of decay and lifetime of transient conformation state, [**SDT**] = 5 eq., [glucose] = 15–2400 eq., GO_*x*_ = 100 U mL^–1^. [**PN–VN**] = 10^–4^ M.

To achieve temporal control over the conformational response of the molecule, the system should have an *in situ* opposite response mechanism with a delay in activation. In our system, it is the oxidation of VN˙^+^ to VN^2+^ that results in reinstating the CT interaction and the subsequent re-folding of the foldamer. This re-folding should re-establish the vesicular morphology with temporal control. Herein, we have achieved this oxidation response *via* two different pathways, which are non-enzymatic and enzymatic oxidation (Fig. 3a and 4a).

Non-enzymatic oxidation involved the use of dissolved oxygen in water to re-oxidize VN˙^+^ to VN^2+^ in a temporal manner to result in transient conformational switching and thereby morphological transition from vesicles to sheets and finally back to vesicles. The rate of formation from vesicles to sheets is governed by reduction and the subsequent return of vesicles from sheets is governed by oxidation. For a transient system, the rate of formation of the transient state should be higher than the rate of decay. In our system, the rate of reduction by **SDT** is instantaneous, hence, to obtain a significant lifetime of the transient state, the oxidation step should be slow. This is possible by employing a mild oxidizing agent such as O_2_. With this, we attempted transient conformational change non-enzymatically and for this a solution of 10^–4^ M **PN–VN** was taken. To the solution 5 equivalents of **SDT** as a chemical fuel were added and an instantaneous increase in absorbance at 900 nm was observed owing to the formation of unfolded **PN–VN˙^+^** assemblies (Fig. 3c).

Monitoring the absorbance at 900 nm over time initially showed a very slow change in absorbance and then a fast decay to nearly zero absorbance, signifying the disappearance of the VN˙^+^ band and thus, the re-folding of **PN–VN** (Fig. 3c). The morphological change which manifested by re-folding was also verified by TEM (Fig. S18b†) which elucidated the retrieval of vesicles. Moreover, since vesicles and sheets differ in size, we observed a change in the DLS from 38 nm to 106 nm and then back to 28 nm which justified temporally controlled chemical fuel-driven conformational and morphological switching (Fig. 3d). To demonstrate the repeatability of the system, refuelling by subsequent addition of **SDT** was carried out and transient cycles were observed to be repetitive with insignificant damping (Fig. 3c). These measurements were done in equilibrated oxygen (open cuvette). The system works with a closed cuvette as well but an open cuvette favours refuelling as a constant amount of deactivator is present (Fig. S19†).

Since the fuel for this transient conformation switching is **SDT**, we then investigated the effect of increasing the equivalents of **SDT**. Upon an increase in the equivalents of **SDT** from 3 to 10, an enhancement in the lifetime of the transient **PN–VN˙^+^** assembly from 10 to 20 minutes was observed. This was because of the presence of a higher amount of fuel which reduces the (re)oxidised VN^2+^ to VN˙^+^ thereby increasing the lifetime that follows a linear trend and hence could be extrapolated to a higher lifetime window (Fig. 3e–f). As a result, we achieved modulation of the lifetime of the transient unfolded conformational state *via* a non-enzymatic redox pathway.

Although a non-enzymatic transient conformational response was obtained with a modular lifetime, control over the rate of oxidation to revert the conformational change was insignificant. To alleviate this, an enzymatic pathway was then employed. In nature, glucose oxidase (GO_*x*_) in the presence of its substrates glucose and oxygen produces hydrogen peroxide (H_2_O_2_) in a controlled pathway for oxidation processes. Inspired by this, we utilized GO_*x*_ in the presence of glucose to synthesize H_2_O_2_ *in situ* which then will oxidize VN˙^+^ to VN^2+^ for the regeneration of the vesicles (Fig. 4a). With this, we investigated the transient conformational switching through an enzymatic pathway.^19^ To a solution of 10^–4^ M **PN–VN**, glucose and GO_*x*_ in an uncapped cuvette, **SDT** was added and the absorbance at 900 nm was monitored over time. Since for a transient system, the lifetime and rate modulation are important parameters to be addressed, we went ahead to study the effect of GO_*x*_ and glucose on the lifetime of the transient state and the rate of the conformational response. At first we varied the units of GO_*x*_ which should directly affect the enzyme kinetics; an increase in the units of GO_*x*_ resulted in a linear change in the lifetime as well as the rate of decay which gives access to a wider temporal regime (Fig. 4b–e).

Furthermore, the glucose equivalents were varied which should also affect the enzyme kinetics. A wide window of glucose equivalents from 15 to 2400 was studied and the effect on the lifetime and rate was investigated at 100 U mL^–1^ of GO_*x*_. The lifetime was observed to decrease from 12 minutes to 5 minutes because of the faster enzyme kinetics (Fig. 4f). A large change in glucose is required for variation in the lifetime, which can be attributed to the fact that we are working at a concentration higher than the Michaelis constant (*K*_m_) value of GO_*x*_ that suggests a low effect of the substrate on the enzyme activity (Fig. S20†).^20^

On comparison of the kinetics of the enzymatic and non-enzymatic pathways at the same concentration of **PN–VN** and **SDT** (Fig. 3c and 4b), two main observations were seen: (i) the lifetimes in the enzymatic pathway were shorter compared to the non-enzymatic pathway and (ii) oxidation of the transient state was much faster in the enzymatic pathway than in the non-enzymatic one. This could be attributed to not only the fact that H_2_O_2_ has a higher oxidation potential than O_2_ but also in the enzymatic pathway both O_2_ and H_2_O_2_ are essentially operating. Although both are operating together, the oxidation potential of H_2_O_2_ qualitatively suggests its larger contribution. Hence, the enzymatic pathway has a higher oxidation rate to oxidize VN˙^+^. Moreover, the H_2_O_2_ produced consumes the excess fuel (**SDT**) thereby decreasing the amount of fuel available in solution. These parameters are compared in Table S1.† Unfortunately, a quantitative distinction could not be achieved for the contributions of all the rates. However, we believe an overall rate constant justifies the above hypothesis. Thus, redox responsive conformational switching with temporal regulation was presented.

## Conclusion

In conclusion, we have shown the translation of a chemical-fuel driven conformational response at a molecular level into temporal switching in a self-assembly. We have categorically analysed the consequences of the conformational change of the molecule on the morphology. Through detailed analysis we have shown that both the enzymatic and non-enzymatic decay pathways can be employed to control the temporal characteristics of the conformational response. We believe that such a design strategy has further applications in building bio-inspired out-of-equilibrium systems for active, adaptive and autonomous materials that are responsive to a multitude of active molecular cues.
