# Supplementary material for: Temporal switching of an amphiphilic self-assembly by a chemical fuel-driven conformational response
Source: Chem Sci. 2017 Jul 11;8(9):6030–6. doi: 10.1039/c7sc01730h (PMC5625291; doi:10.1039/c7sc01730h)
Supplement: Supplementary file 1 [file SC-008-C7SC01730H-s001.pdf]

Electronic Supplementary Material (ESI) for Chemical Science.
This journal is © The Royal Society of Chemistry 2017

Supporting Information

Temporal Switching of an Amphiphilic Self-assembly by a Chemical Fuel-Driven Conformational Response

Krishnendu Jalani, Shikha Dhiman, Ankit Jain,* Subi J. George*

Supramolecular Chemistry Laboratory, New Chemistry Unit, Jawaharlal Nehru Centre for Advanced Scientific Research (JNCASR), Jakkur, Bangalore, India-560064

Table of Contents

1. General methods
2. Experimental section
3. Synthetic schemes and procedures
4. Packing factor calculation
5. Supporting figures
6. References

1. General Methods

NMR Measurements: NMR spectra were recorded with a Bruker AVANCE 400 (400 MHz) Fourier transform NMR spectrometer with chemical shifts reported in parts per million (ppm) with respect to TMS. Splitting patterns are designated as s, singlet; d, doublet; bs, broad singlet; m, multiplet; t, triplet.

Optical Measurements: UV-Visible absorption spectra were recorded on a Perkin Elmer Lambda 900 UV-Vis-NIR Spectrometer and emission spectra were recorded on Perkin Elmer LS 55 Luminescence Spectrometer. Fluorescence spectra of solutions were recorded with 320 nm excitation wavelength. 10 mm x 10 mm or 10 mm x 2 mm quartz cuvettes were used for measurements.

High Resolution Mass Spectrometry (HRMS): High Resolution Mass Spectra (HRMS) were recorded on an Agilent 6538 Ultra High Definition (UHD) Accurate-Mass Q-TOF-LCMS system using electrospray ionization (ESI) technique either in positive mode or negative mode.

Transmission Electron Microscopy (TEM): TEM measurements were performed on JEOL JEM 3010 operated at 300 kV. Samples were prepared by placing a drop of solution on carbon coated copper grids followed by drying at room temperature. The images were recorded with an operating voltage of 300 kV. In order to get a better contrast, the samples were stained with uranyl acetate (1 wt % in water) before the measurements.

Cryogenic-TEM (Cryo-TEM): For Cryo-TEM measurement, samples were prepared using FEI Vitrobot system by plunge freezing in liquid ethane to preserve the native structure. Following are the parameters that were used: Blot time (s) - 1.0, Blot force - 0, Wait time (s) - 1.0, Blot total - 1, Drain time (s) - 0.5. The temperature was maintained at 22 °C and humidity was >90. Holey carbon grids were used for sample preperation, bought from EMS. Imaging was done using Tecnai G2 Spirit Bio-TWIN Transmission Electron microscope at 100 kV.

Atomic Force Microscopy (AFM): AFM measurements were performed on a Veeco di Innova SPM operating in tapping mode regime. Micro-fabricated silicon cantilever tips doped with phosphorus with frequency between 235 and 278 kHz and a spring constant of 20-40 Nm^{-1} were used. The samples were prepared by drop casting the solution on glass substrate and dried in air followed by vacuum drying at room temperature.

Dynamic Light Scattering experiments (DLS): The measurements were carried out using a NanoZS (Malvern UK) employing a 532 nm laser at a back scattering angle of 173°.

Zeta potential measurement: Zeta potential measurements were carried out using a Nano ZS (Malvern UK).

Confocal microscopy: Confocal microscopy imaging was done at room temperature using a Zeiss LSM 510 META laser scanning confocal microscope with a laser excitation of λ_{exc} = 320 nm. The microscope objective of 63X (NA 1.4) and 100X (NA 0.5) were employed. Samples were prepared by dropping the solution on a glass slide and measurements were done in liquid state by following the standard procedure.

2. Experimental section

Materials: Sodium dithionite, Cucurbit[7]uril, Adamantyl amine, Glucose Oxidase enzyme (GOx) and glucose were purchased from commercial suppliers. All the chemicals were used directly without any further purification. The water used in all experiments was of Millipore Milli-Q grade.

Protocol for passive conformational change: The UV-Vis spectroscopic measurements were done in capped quartz cuvette. Stock solutions were prepared such that there is minimum dilution. For measurements, in a cuvette, a solution of 10^{-4} M **PN-VN** was taken at room temperature, to it appropriate equivalents of CB[7] were added and measurements were done. Then, required equivalents of ADA were added and measurement was continued.

Protocol for transient conformational change: All the measurements were done at pH 7 aqueous buffer unless mentioned otherwise. All the UV-Vis spectroscopic measurements were done in uncapped quartz cuvette (except when stated) for equilibration of O_2 in solution and at continuous stirring condition for homogeneity. Freshly prepared sodium dithionite (**SDT**) stock was used for each measurement as it is unstable in water. Stock solutions were prepared such that there is minimum dilution.

1. For non-enzymatic conformational change: Measurements were done at room temperature.
   In a cuvette, a solution of 10^{-4} M **PN-VN** was taken, required amount **SDT** was added and measurements were done.
2. For enzymatic conformational change: Temperature was maintained at 35°C for better enzymatic activity.
   In a cuvette, a solution of 10^{-4} M **PN-VN** with required amount of glucose and Gox were taken, to it appropriate amount of **SDT** was added and measurements were done immediately.

3. Synthetic schemes and procedures

Materials: Pyranine, carbon tetrabromide, triphenylphosphine, hexaethylene glycol, 4,4'-bipyridine, 1-bromododecane and N,N-diisopropyl ethylamine, were purchased from commercial suppliers. All the chemicals were used directly without any further purification.

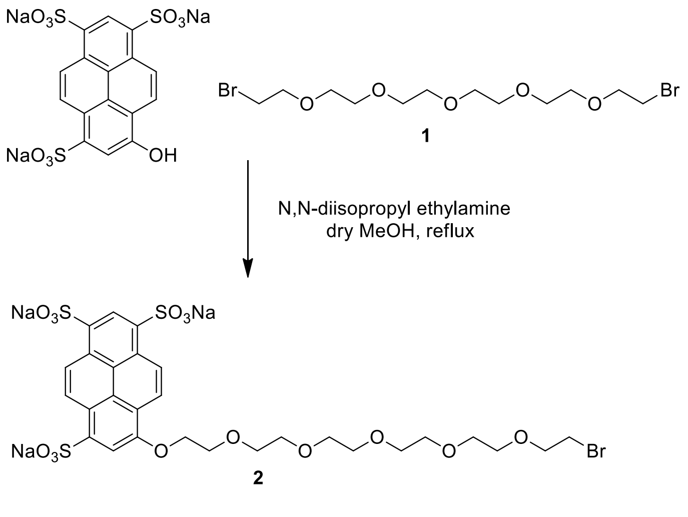

Scheme S1. Synthetic route for the molecule **2.**

Synthesis of **1**: The molecule **1** was synthesized following the reported procedure.[S1a]

Synthesis of **2**: Pyranine (1.79 g, 3.42 mmol) with excess of **1** (5.1 g, 12.05 mmol) was taken in methanol (80 ml) and to that diisopropyl ethylamine (2.5 ml, 15.08 mmol) was added and continued stirring at 65 °C for 8 days. The reaction was monitored by TLC in 50% CH_3OH in $CHCl_3$.The mixture was then concentrated under vacuum and the compound was purified by silica gel column chromatography with 40% CH_3OH in $CHCl_3$ to give viscous yellow liquid product in 10% yield. ^1H NMR (400 MHz, CD_3OD, TMS): δ (ppm) 9.40 (s, 1H), 9.26 (d, J =

9.6 Hz, 1H), 9.22 (d, *J* = 9.6 Hz, 1H), 9.14 (d, *J* = 10 Hz, 1H), 8.70 (d, *J* = 9.6 Hz, 1H), 8.44 (s, 1H), 4.61 (t, 2H), 4.11 (t, 2H), 3.83 (t, 2H), 3.37 (t, 2H), 3.56 (m, 16H). ^{13}C NMR (100 MHz, CD_3OD) δ (ppm) 164.88, 154.24, 142.40, 138.56, 138.41, 131.20, 130.74, 128.50, 127.23, 127.15, 126.87, 126.43, 124.92, 123.73, 122.70, 122.52, 72.23, 71.96, 71.58, 71.52, 71.38, 71.28, 71.17, 70.90, 70.25, 55.82, 31.47. HRMS: *m/z* calculated: $C_{29}H_{32}BrO_{16}S_3$: 783.0092, found: 783.0133 $[M + 2H]^-$.

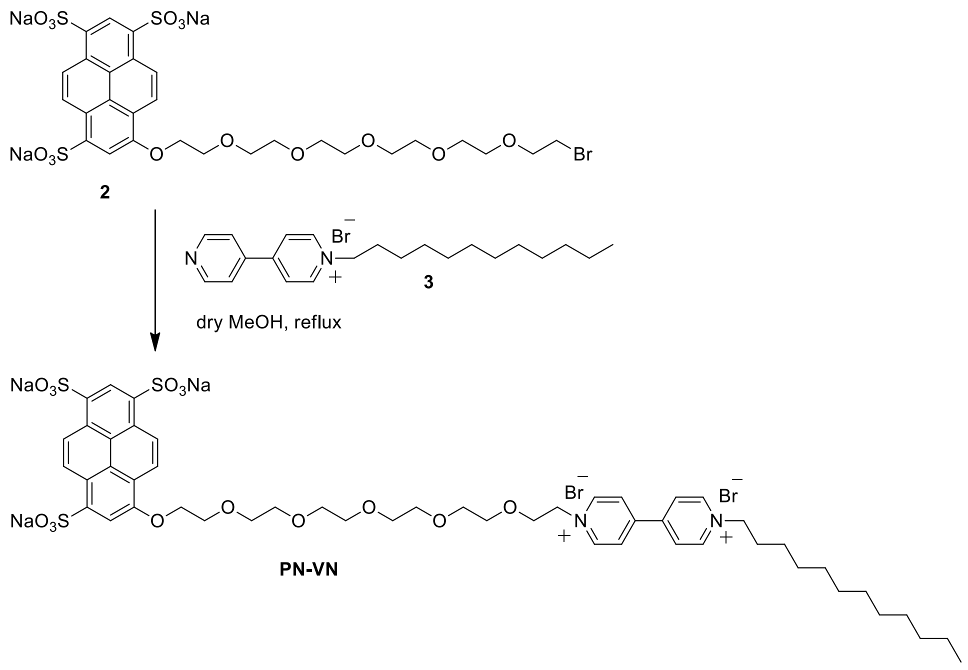

Scheme S2. Synthetic route for **PN-VN.**

Synthesis of **3**: The molecule **3** was synthesized following the reported procedure.[S1b]

Synthesis of **PN-VN**: Molecule **2** (0.3 g, 0.35 mmol) and **3** (0.47 g, 1.4 mmol) were mixed in 40 ml of dry MeOH and continued stirring at 75 °C for 5 days. The mixture was then concentrated under vaccum and purified by silica gel column chromatography (40% MeOH in $CHCl_3$) to give red colour amorphous solid product in 15% yield. ^{1}H NMR (400 MHz, CD_3OD, TMS): δ (ppm) 9.17 (s, 1H), 9.03 (d, *J* = 9.6, 1H), 8.91 (d, *J* = 10.4, 2H), 8.63 (d, *J* = 7.2, 2H), 8.55 (d, *J* = 6.8, 2H), 8.34 (d, *J* = 9.6, 1H), 8.15 (s, 1H), 7.51 (d, *J* = 6.8, 2H), 7.46 (d, *J* = 6.8, 2H), 4.69 (t, 2H), 4.59 (t, 2H), 4.52 (t, 2H), 4.11-3.21 (m, 20H), 2 (m, 2H), 1.38 -1.28 (m, 18H), 0.88 (t, 3H). ^{13}C NMR (100 MHz, CD_3OD) δ (ppm) 153.97, 147.52, 147.32, 147.04, 146.36, 142.69, 138.79, 138.55, 130.46, 130.15, 128.42, 126.82, 126.54, 126.42, 126.34, 125.97, 125.27, 124.89, 121.84, 110.54, 71.97, 71.70, 71.63, 71.55, 71.24, 70.85, 70.26, 69.88, 63.31, 62.87, 33.07, 32.31, 30.74, 30.64, 30.54, 30.46, 30.11, 27.25, 23.72, 14.43. HRMS: *m/z* calculated: $C_{50}H_{65}N_2O_{15}S_3$: 1029.3547, found: 1029.3522 $[M+2H]^+$.

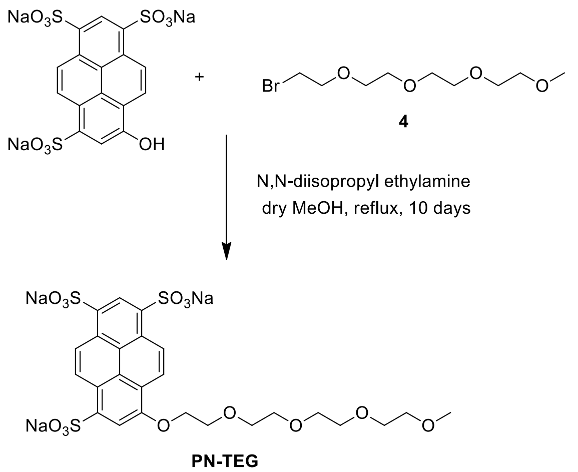

Scheme S3. Synthetic route for **PN-TEG**

Synthesis of monomethyl tetraethylene glycol (**4**): The molecule **4** was synthesized following the reported procedure.[S2]

Synthesis of **PN-TEG**: Pyranine (1.10 g, 2.1 mmol) and N,N-diisopropyl ethylamine (0.70 g, 5.4 mmol) were added to **4** (1.8 g, 7.4 mmol) in 15 ml of dry MeOH. The resulting mixture was refluxed with stirring for 10 days. The solvent was then evaporated and the compound was purified by precipitation in ethanol followed by silica-gel column chromatography in 35% to 55% MeOH/$CHCl_3$ in gradient to give pale yellow amorphous solid product in 20% yield. ^{1}H NMR (400 MHz, $CDCl_3$): δ = 9.19 (s,1H), 9.16 (d, *J* = 9.6 Hz, 1H), 9.07 (d, *J* = 9.6 Hz, 1H), 8.99 (d, *J* = 10.0 Hz, 1H), 8.91 (d, *J* = 9.6 Hz, 1H), 8.43 (s,1H), 4.15 (t, 2H), 3.83 (t, 2H), 3.70 (t, 2H), 3.63 (t, 2H), 3.42 (t, 2H), 3.16 (t, 2H), 3.07 (t, 2H), 2.96 (m, 5H). ^{13}C NMR (100 MHz, CD_3OD) δ (ppm) 154.20, 142.18, 138.11, 131.24, 130.78, 128.46, 127.19, 127.12, 126.74, 126.34, 124.81, 123.74, 122.75, 122.54, 111.04, 72.72, 71.90, 71.54, 71.44, 71.31, 71.11, 70.89, 70.24, 59.02. HRMS: *m/z* calculated: $C_{25}H_{27}O_{14}S_3$: 647.0568, found: 647.0680 $[M+2H]^-$.

4. Packing factor calculation:

The packing factor[S3, S4] was calculated from the following equation S-1

$$\boldsymbol{f} = \boldsymbol{v}/\boldsymbol{al} \quad \text{(S-1)}$$

where $\boldsymbol{f}$ is packing factor, $\boldsymbol{l}$ is the hydrophobic chain length, $\boldsymbol{a}$ is the ratio of hydrophilic to hydrophobic interfacial area.

For an amphiphile with hydrophobic alkyl chain, the hydrophobic chain length ($\boldsymbol{l}$) and hydrophobic volume ($\boldsymbol{v}$) can be calculated by using the following Tanford equations[S5]:

$$\boldsymbol{v} = 27.4 + 26.9\,\boldsymbol{n} \quad \text{(S-2)}$$

$$\boldsymbol{l} = 1.5 + 1.265\,\boldsymbol{n} \quad \text{(S-3)}$$

where, $\boldsymbol{n}$ corresponds to the number of carbon atoms in the alkyl chain.

The ratio of hydrophilic to hydrophobic interfacial area ($\boldsymbol{a}$) was calculated as

$$\boldsymbol{a} = \boldsymbol{\pi}\left(\frac{\boldsymbol{d}}{\mathbf{2}}\right)^{\mathbf{2}} \quad \text{(S-4)}$$

where $\boldsymbol{d}$ corresponds to the diameter of the interfacial area.

Then using the above equation (S-2) and (S-3), the hydrophobic volume for C12 alkyl chain of the **PN-VN** was calculated to be $\boldsymbol{v}$ = 350 Å^3 and length $\boldsymbol{l}$ = 16.68 Å.

Packing factor calculation for folded PN-VN (Scheme S4)**:**

Using wB97x-D/6-31G+(d,p) level of theory in gas phase, the structure was optimized (Scheme S4) to get average diameter of hydrophobic-hydrophilic interfacial area of 6.27 Å.

a was calculated as

$$\boldsymbol{a} = \boldsymbol{\pi}\left(\frac{\boldsymbol{d}}{\mathbf{2}}\right)^{\mathbf{2}} = \boldsymbol{\pi}\left(\frac{\mathbf{6.27}\,\text{Å}}{\mathbf{2}}\right)^{\mathbf{2}} = 30.88\ \text{Å}^2$$

$$\boldsymbol{f} = \boldsymbol{v}/\boldsymbol{al} = \frac{\mathbf{350}\ \text{Å}^{\mathbf{3}}}{(\mathbf{30.88}\ \boldsymbol{x}\ \mathbf{16.68})\text{Å}^{\mathbf{3}}} = 0.68$$

which matches well with the packing factor of vesicles 0.5 to 1 with bilayer packing.[S4]

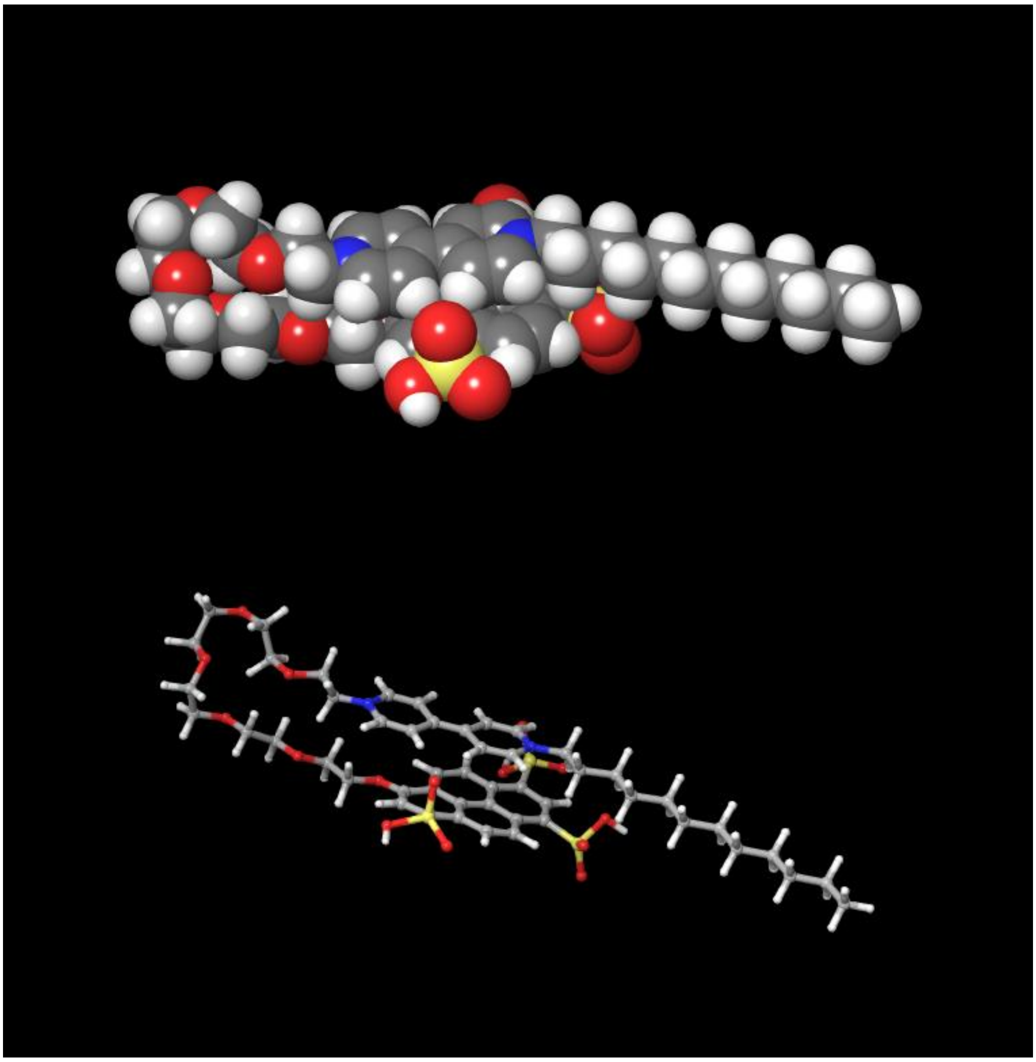

Scheme S4: The molecular structure of **PN-VN** in the native folded state was obtained from the optimized molecular model generated with wB97x-D/6-31G + (d,p) level of theory in gas phase.

Packing factor calculation for unfolded PN-VN with CB[7] (Scheme S5a)**:**

Using the diameter of the carbonyl portal area [S6] of CB[7] = 5.4 Å.

a was calculated as

$$\boldsymbol{a} = \boldsymbol{\pi}\left(\frac{d}{2}\right)^{2} = \boldsymbol{\pi}\left(\frac{5.4\,\text{Å}}{2}\right)^{2} = 22.89\ \text{Å}^{2}$$

$$\boldsymbol{f} = \boldsymbol{v}/\boldsymbol{al} = \frac{350\ \text{Å}^{3}}{(22.89\ x\ 16.68)\text{Å}^{3}} = 0.91$$

which matches with the packing factor of sheets ~1 with lamellar packing. [S4]

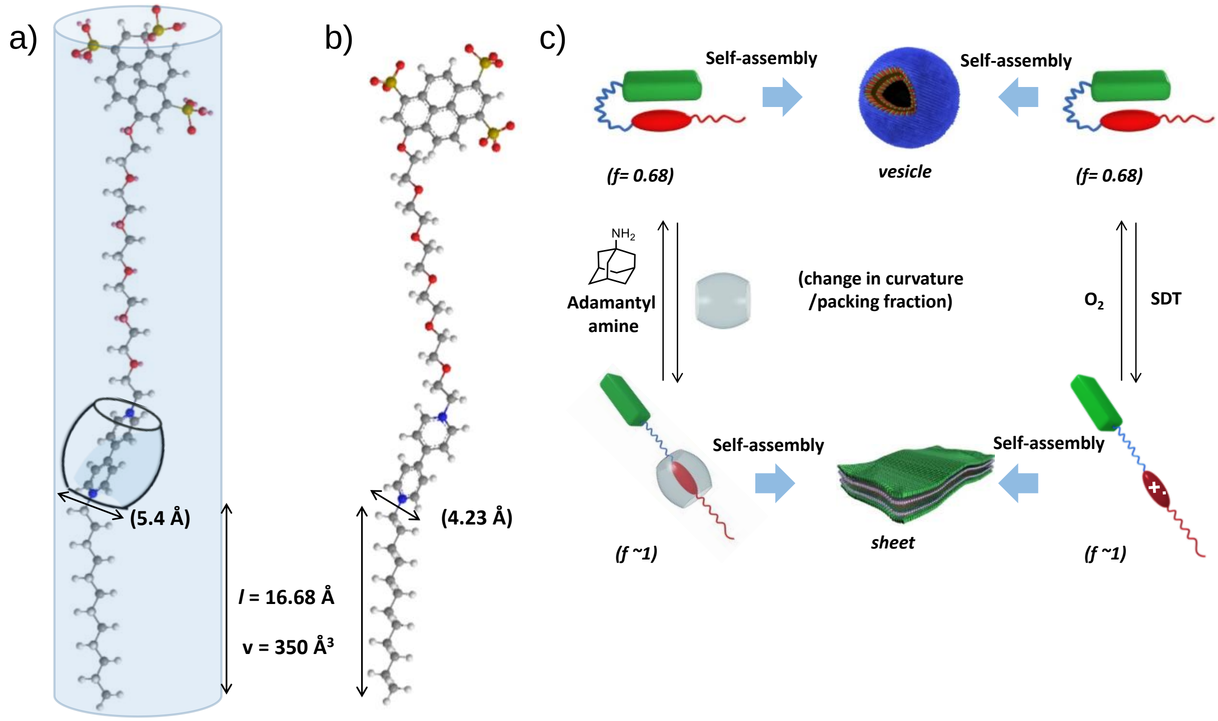

Scheme S5: Packing factor calculation for unfolded **PN-VN**. Schematic representation for the molecular structure of **PN-VN** molecule in the unfolded state. a) with CB[7], b) without CB[7]. The molecular structure of **PN-VN** was obtained from the energy minimized molecular model generated with Chem 3D Pro v.10 software.[S7] c) Schematic representation depicting the reversible conformational switching of the foldamer (**PN-VN**) with change in packing factor (***f***) resulting in the morphological variation.

Calculation of the packing factor (*f*) for unfolded PN-VN without CB[7] (Scheme S5b)**:**

The calculation of packing factor of the unfolded **PN-VN** was done in similar method as of **PN-VN** with CB[7] by using the equations S-1 to S-4. Without CB[7] the diameter of of hydrophilic to hydrophobic surface area (***d***) was obtained as 4.23 Å (obtained from Chem 3D Pro v.10 software) which is very close to 5.4 Å, the CB[7] carbonyl portal diameter. The packing factor was calculated to be ***f*** ~1, which suggests lamellar packing for sheet morphology.

Using the diameter of the interfacial area of molecule = 4.23 Å.

a was calculated as

$$\boldsymbol{a} = \boldsymbol{d}^2 = 4.23\text{Å}^2 = 17.89\ \text{Å}^2$$

$$\boldsymbol{f} = \boldsymbol{v}/\boldsymbol{al} = \frac{350\ \text{Å}^3}{(17.89\ x\ 16.68)\text{Å}^3} \sim 1$$

which matches with the packing factor of sheets ~1 with lamellar packing.[S4]

5. Supporting figures:

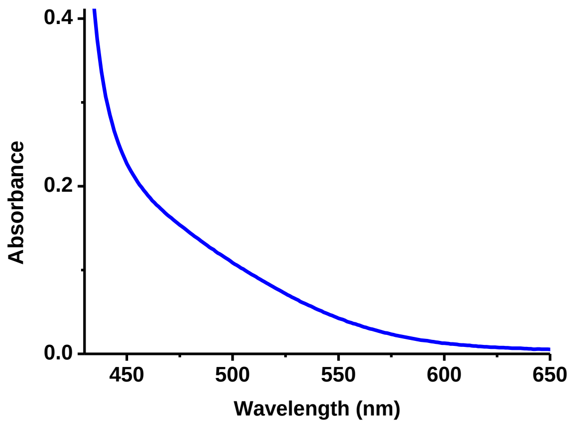

Figure S1. UV-vis spectra of the **PN-VN** foldamer in H_2O with the charge-transfer (CT) band at 480 nm, suggesting the pre-folded conformation via pyranine-viologen CT interactions, ([**PN-VN**] = 10^{-4} M).

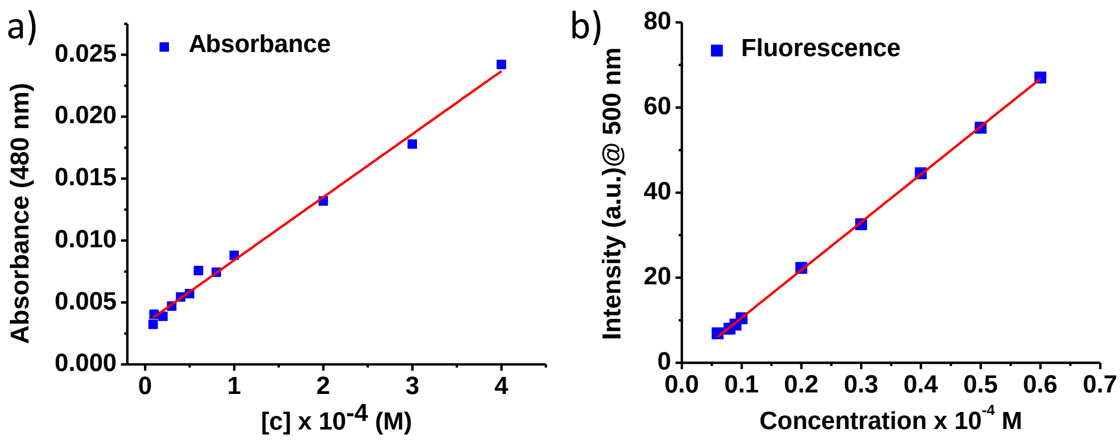

Figure S2. a) The linear dependence of the CT absorbance at 480 nm of the **PN-VN** foldamer with concentration suggesting the intramolecular CT interaction in the foldamer. The dilution experiment was performed in the concentration regime of $4x10^{-4}$ M to $6x10^{-6}$ M. b) The concentration dependent emission changes of the **PN-VN** foldamer was plotted monitoring 500 nm band which shows linear dependence of emission upto $6x10^{-6}$ M, the emission below this concentration could not be plotted because of the quenched emission of the folded state.

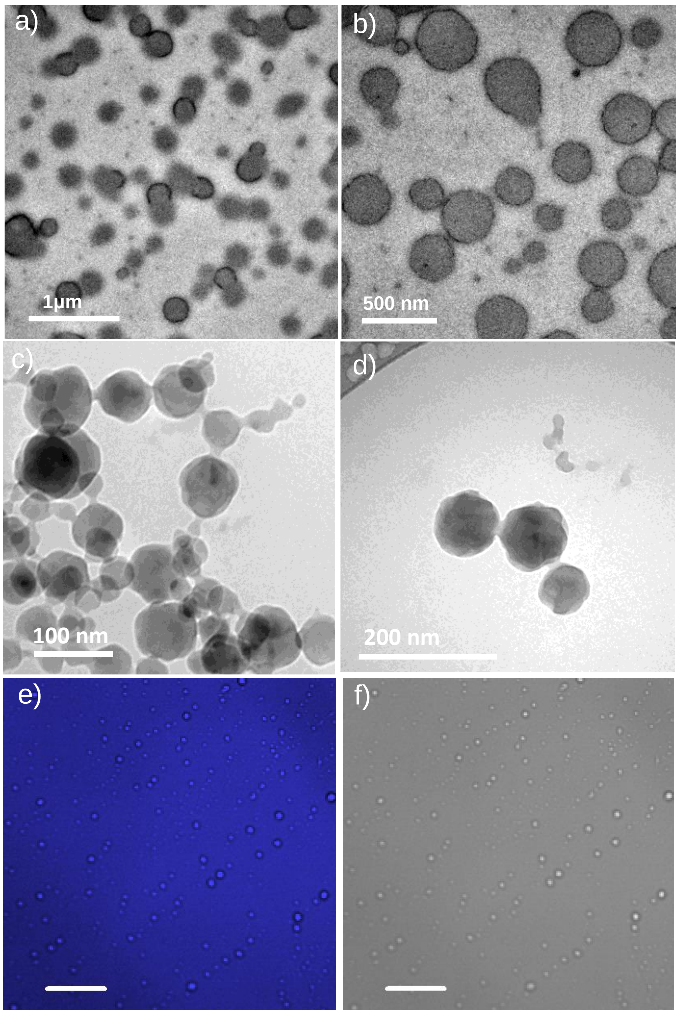

Figure S3. a), b) TEM, c), d) Cryo-TEM, and e), f) confocal fluorescence microscopic and corresponding bright field (scale bar: 20 μm) images for the **PN-VN** vesicles (c=10^{-4} M).

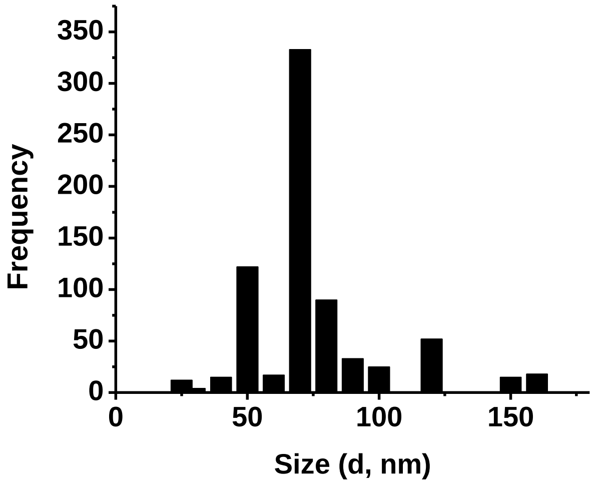

Figure S4. Histogram corresponding to the size of the vesicles in water obtained from the inspection of 749 vesicles in the cryo-TEM images which were taken from different regions of the cryo-TEM grid. Average size = 75 nm and Standard deviation= 44. (d = diameter of the vesicles, error bar in size: ± 5 nm).

Calculation of the CT association constant of the PN-VN foldamer:

As the CT absorbance of **PN-VN** involves scattering above CAC, the association constant could not be calculated with relaibility. We further attempted to calculate the intramolecular CT association constant of **PN-VN** using concentration dependent spectroscopic measurements and isothermal titration calorimetry (ITC), however due to difficulties no reliable value was obtained and hence we restrict ourselves to obtain a lower limit of association constant (K_{PN-VN} ~10^5) using model compounds **PN-TEG** and **DMV** that undergo intermolecular charge transfer (*vide infra*). Further, the concentration dependent emission studies (Figure S2b) of **PN-VN** foldamer shows a linear dependence till the low concentration of $6x10^{-6}$ M reiterating the lower limit of association constant of the order of 10^5. Moreover, the study presented here doesn't rely on absolute values of association constants, thus doesn't affect the quality of study.

The association constants of the **PN-TEG** and **DMV** intermolecular CT pair was calculated using the following two methods.

Method 1.

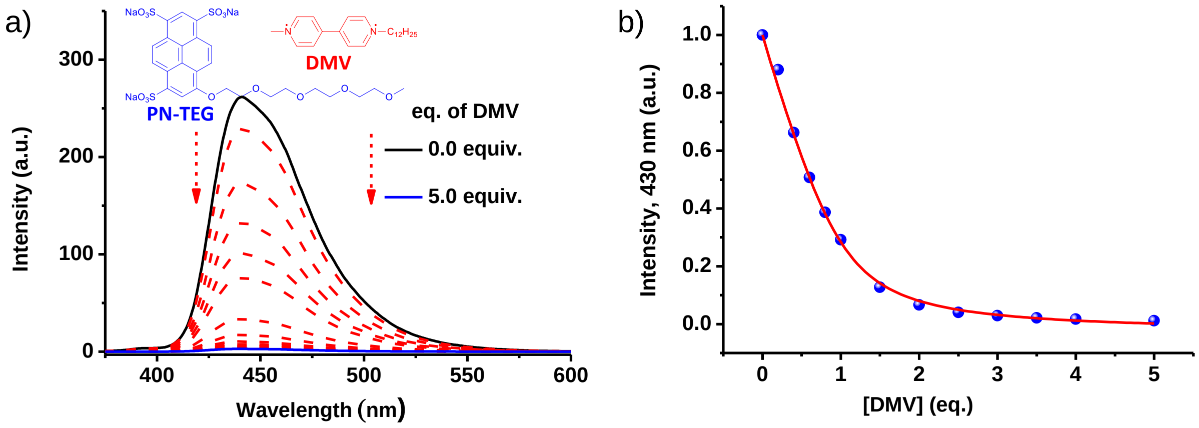

Figure S5. a) Emission spectra of **PN-TEG** on titration with **DMV** molecule and b) Plot of emission intensity of **PN-TEG** at 430 nm versus eq. of **DMV** and the nonlinear least-squares curve-fitting with equation S-5 to determine the binding constant ([**PN-TEG**] = 10^{-4} M).

Note: b) Association constant (K_a) between **PN-TEG** and **DMV** was calculated by fitting their titration using Equation S-6, (mentioned below).[S8] The K_a was calculated to be $7.4x10^4$ M^{-1}. It was found that around 3.5 eq. of **DMV** was required to result the emission intensity of **PN-TEG** equal to **PN-VN** emission intensity in its folded state. Hence, we believe that for the foldamer **PN-VN,** association constant would be of the order of ~10^5 (**DMV**: dodecyl methyl viologen).[S9]

$$\Delta F_{obs} = k_{\Delta HG} \frac{\left(H_0 + G + \frac{1}{K_a}\right) + \sqrt{(H_0 + G_0 + \frac{1}{K_a})^2 - 4H_0G_0}}{2} \quad \text{.....(S-6)}$$

ΔF is the fluorescence changes at 450 nm, H_0 = [**PN-TEG**] = 10^{-4} M, G_0 = [**DMV**] = 10^{-4} M, K_a is the binding constant between **PN-TEG** and **DMV** and $k_{\Delta HG}$ (response factor). It was assumed that the fluorescence change was in a linear relationship with the concentration of donor-acceptor complex in the solution. In this case, concentration of the **PN-TEG** was fixed to 10^{-4} M and the fluorescence intensity ΔF_{obs} at 450 nm was fitted to Equation S-5 using nonlinear least-squares curve-fitting method. The binding constant (K_a) between the **PN-TEG** and **DMV** was calculated to be 7.4 x 10^4 M^{-1}.

Method 2.

The intermolecular association constant of the **PN-TEG** and **DMV** CT pair was also calculated by using another equation with the fitting of same fluorescence titration data shown below.

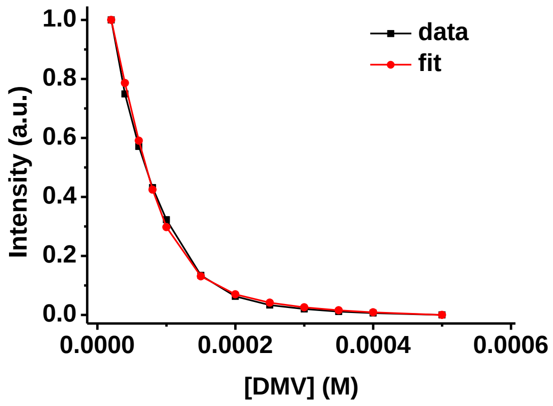

Figure S6. Plot of emission intensity of **PN-TEG** at 430 nm versus concentration of **DMV** and the nonlinear curve-fitting to determine the intermolecular binding constant K_a = 1.0x10^5 ([**PN-TEG**] = 10^{-4} M).

The equation used for fitting S-7

$$\mathrm{G} + \mathrm{H} \rightleftharpoons \mathrm{GH}$$

$$\mathrm{K} = \frac{K_a}{K_d} = \frac{[GH]}{[G][H]}$$

$$[G]_t = [G] + [GH]$$

$$[H]_t = [H] + [GH]$$

$$\theta_{GH} = \frac{[GH]}{[G_t]} = \frac{K[H]}{1+K\,[H]} =$$

$$\frac{2K[H]t}{(1 + \mathrm{K}\,[G]_t + \mathrm{K}[H]_t + \sqrt{K^2[G]_t{}^2 + 2K[G]_t - 2K^2[G]_t\,[H]_t + 1 + 2K[H]_t + K^2[H]_t{}^2}} \text{...(S-7)}$$

Where K is the equilibrium binding constant for a host-guest binding event. The host concentration is denoted as [H] = [**PN-TEG**] and guest concentration is denoted as [G] = [**DMV**]. The total guest concentration $[G]_t$ is the sum of the unbound guest concentration [G] plus the concentration of guest-host complex [GH]. This mass balance is equally valid for host as well. Therefore the fraction of bound guest molecule θ_{GH} is the ratio of [GH] and $[G]_t$. The equation was derived as follows.

The equation was used for the fitting of fluorescence titration data of the intermolecular binding event of **PN-TEG** and **DMV** which resulted the association constant as K = 1.0x10^5.

The calculation using above equation suggest an intermolecular CT of order 10^4-10^5 in consistence with reported value for pyranine-viologen.[S10] Hence, we believe that the intramolecular association constant of **PN-VN** foldamer would be greater than 10^5 due to high local concentration compared to intermolecular system.

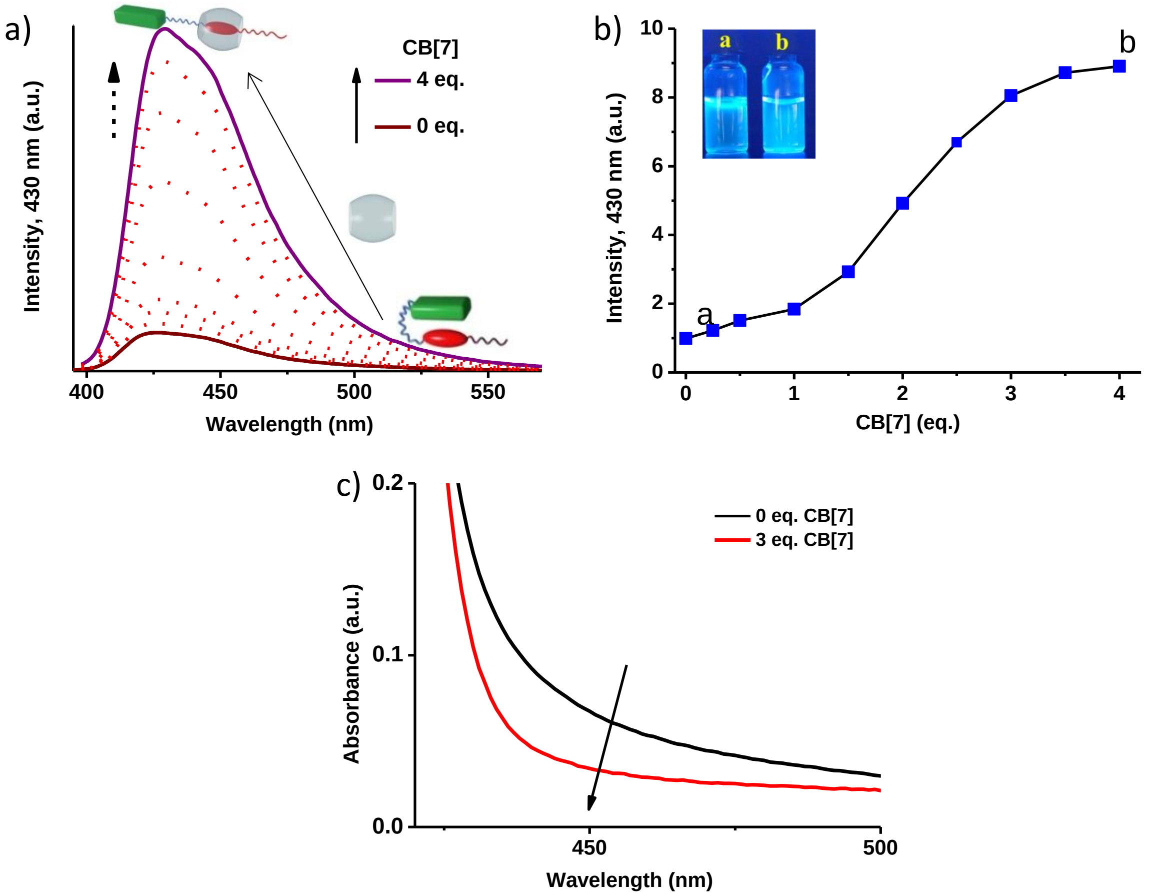

Figure S7. a) Emission spectra of **PN-VN** in water upon titration with CB[7]. b) Plot of **PN-VN** emission intensity at 430 nm vs. equivalent addition of CB[7], Inset of b) show the photographs of **PN-VN** (left) and **PN-VN** + CB[7] (1:4) (right) solutions under 365 nm UV irradiation. c) Decrease in charge transfer band on addition of CB[7]. ([**PN-VN**] = 10^{-4} M, CB[7] = 0 to $5x10^{-4}$ M, λ_{exc} = 320 nm).

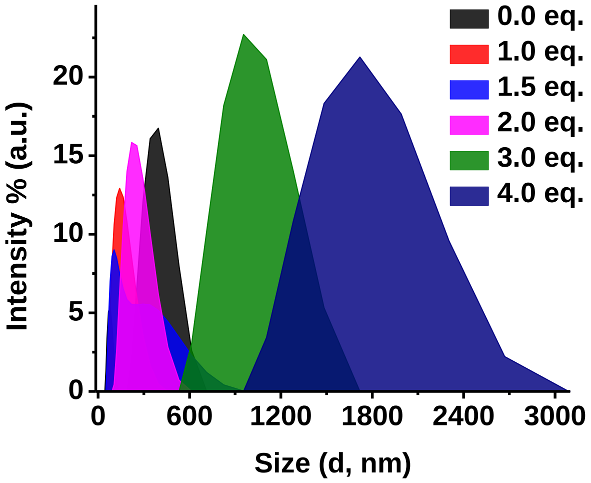

Figure S8. Intensity distribution corresponding to the change in DLS size for the vesicle to sheet transformation with addition of CB[7] to **PN-VN**. ([**PN-VN**] = 10^{-4} M, H_2O), d: diameter of the vesicles in nm scale.

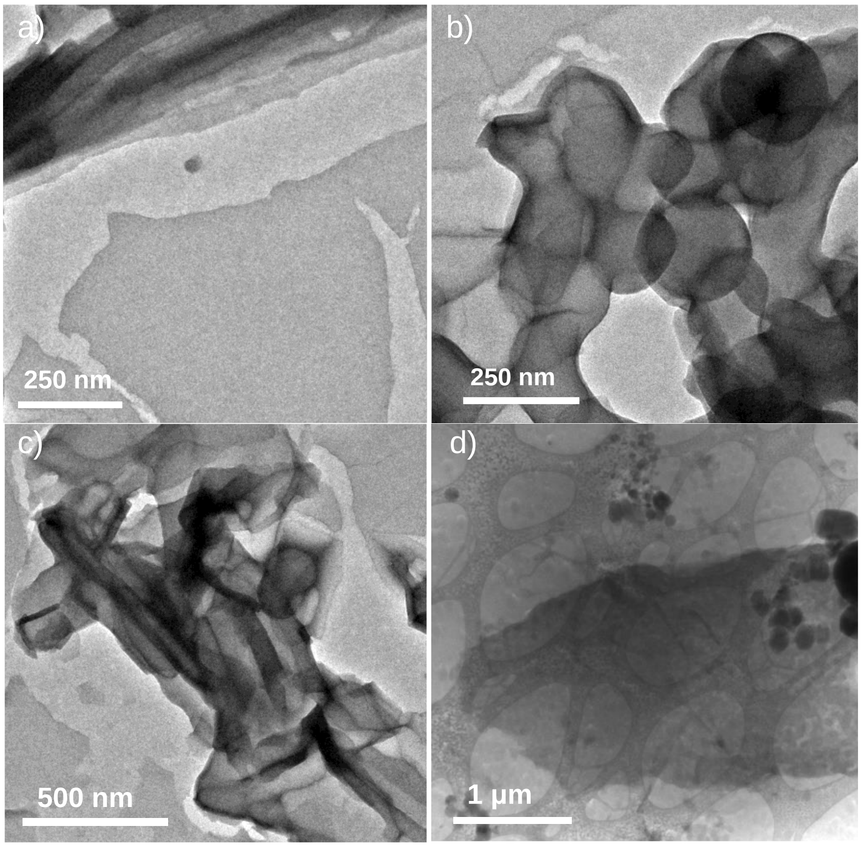

Figure S9. a), b), c) TEM and d) Cryo-TEM images of the 2D sheets of the unfolded conformation of **PN-VN** (10^{-4}M), in presence of 4 eq. of CB[7].

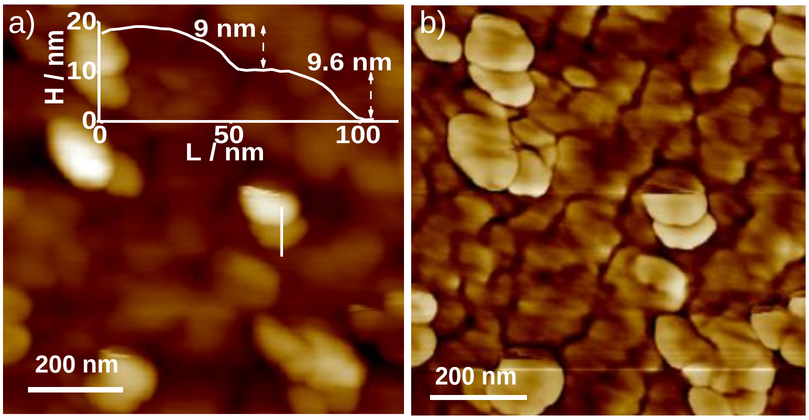

Figure S10. a) and b) are the AFM height and phase images of the sheet for **PN-VN** with the addition of 4 eq. CB[7]. Height profile in the inset of a) shows height of sheets in 9.0 - 9.5 nm range, suggesting that amphiphilic bilayers of the unfolded molecules are organized perpendicular to the 2-D sheet. **PN-VN** (10^{-4}), CB[7] = 4 eq.

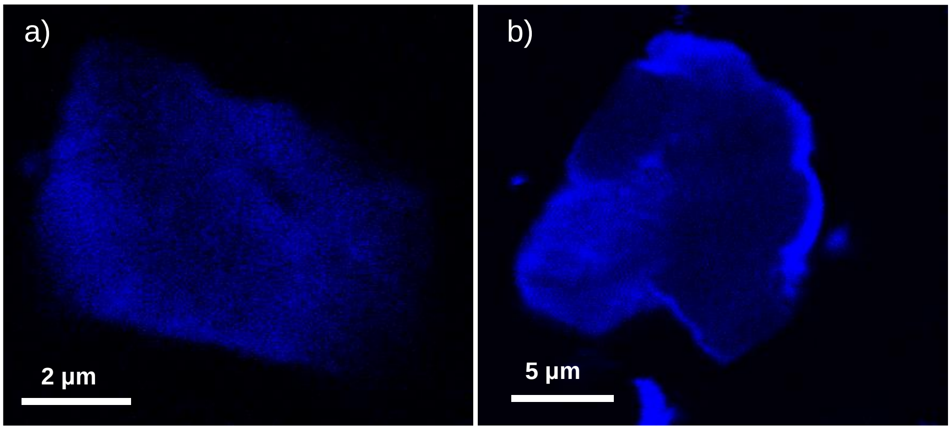

Figure S11. Confocal fluorescence microscope images of the sheet after addition of 4 eq. of CB[7] to **PN-VN**. [**PN-VN**] = 10^{-4} M, H_2O.

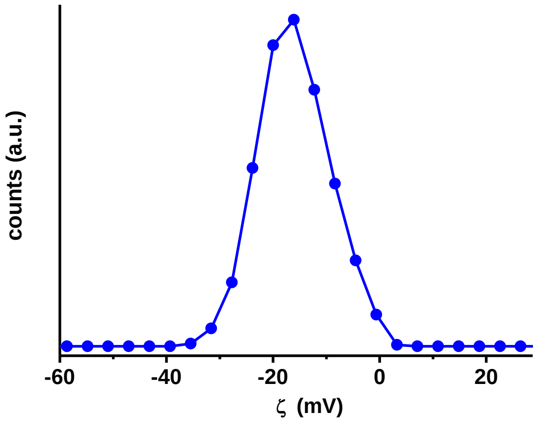

Figure S12. Zeta potential of the **PN-VN** + CB[7] sheets originating from the negatively charged pyranine sulphonate groups on the sheet surface ([**PN-VN**] = 10^{-4} M, CB[7] = 4 eq., H_2O).

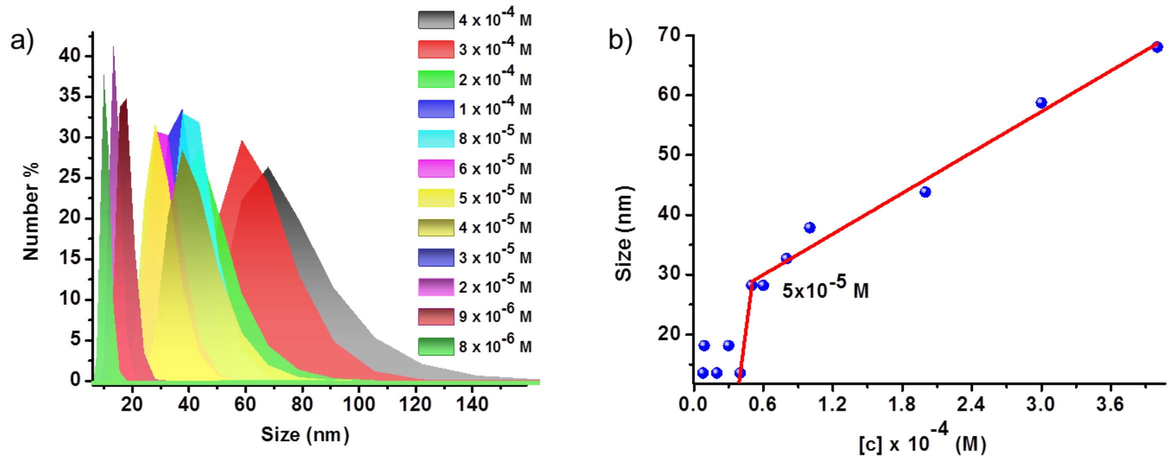

Figure S13. a) DLS spectra for the concentration dependent studies of **PN-VN** to determine the CAC of the foldamer vesicles. b) Plot of DLS size vs concentration showing the CAC of 5x10^{-5} M.

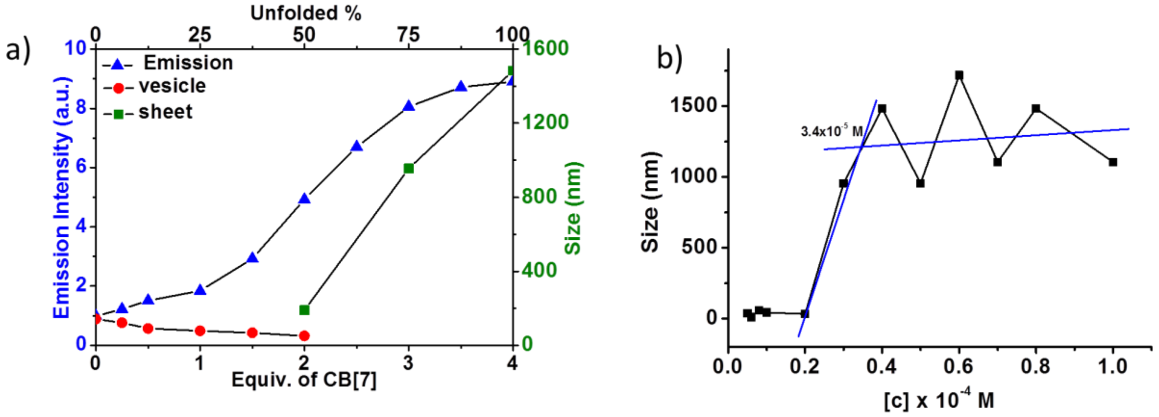

Figure S14. a) Plot of DLS size and emission intensity vs. equivalent of CB[7] and unfolded percentage of foldamer. b) Plot of DLS size vs concentration for the dilution dependent studies of **PN-VN** + 4 eq. CB[7] assembly showing the CAC of the sheets as 3.4 x10^{-5} M.

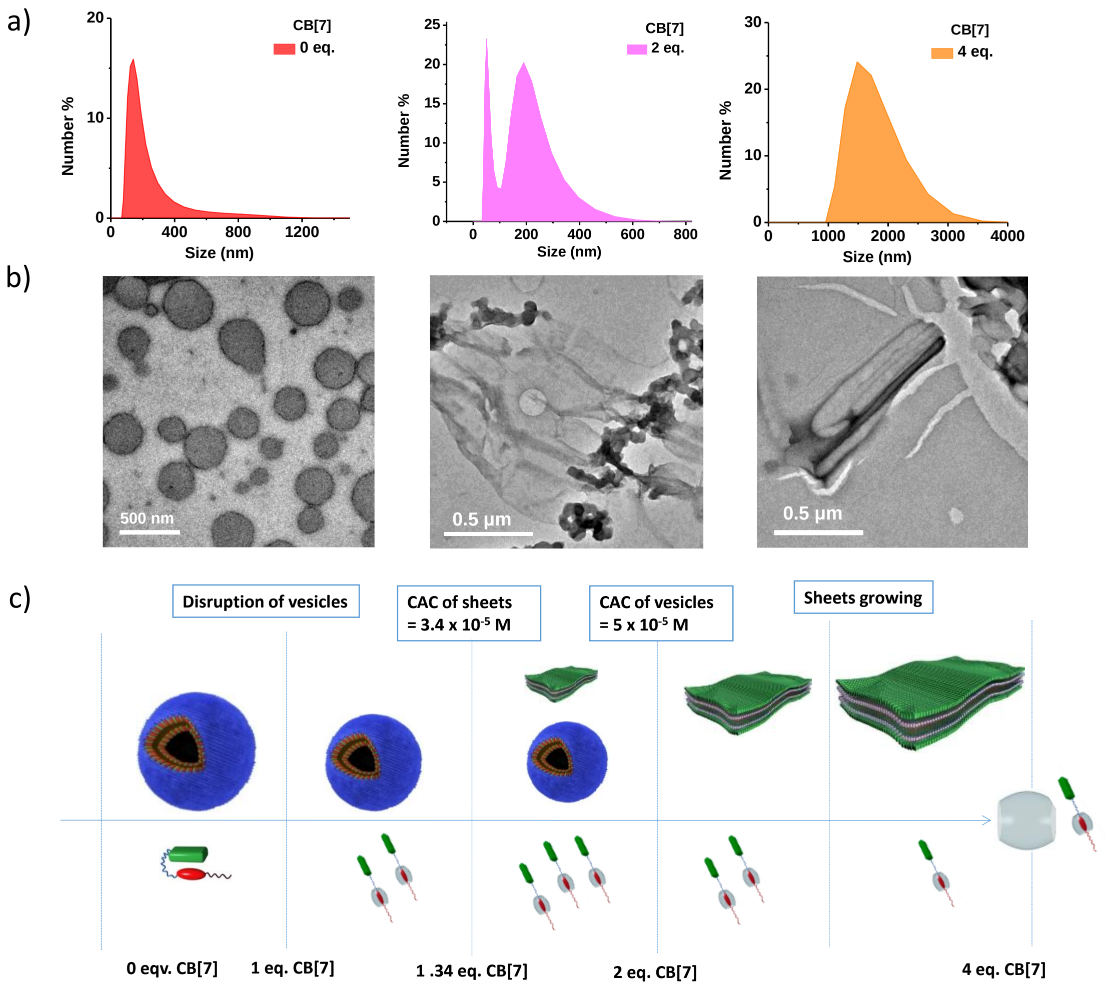

Figure S15. a) Plot of DLS size at different equivalents of CB[7] and b) their corresponding TEM images. c) Proposed schematic representation for vesicle to sheet transformation.

Note: To get a detailed insight into vesicle to sheet transformations during **PN-VN** titration with CB[7], we have probed fluorescence, DLS and TEM experiments at different stages during the titration. Fluorescence titrations show a sigmoidal trend with addition of CB[7], where around 2 eq. of CB[7] a steep increase in emission was observed (Figure S14a). Also, DLS experiments show that on addition of CB[7] there is an initial decrease in size till 2 eq. which are vesicles in morphology as visualised by TEM. This can be attributed to decrease in the concentration of the folded conformations with increasing eq. of CB[7]. Detailed characterization at 2 eq. of CB[7] showed a bimodal distibution suggesting the co-existence of vesicles and sheets, which is further supported by the TEM. Hence sheets starts growing once the concentration of the unfolded monomers reaches its CAC for the formation of sheets. On further increase of CB[7] the concentration of folded monomers goes below the CAC of vesicles resulting in a DLS characteristic of the sheets only.

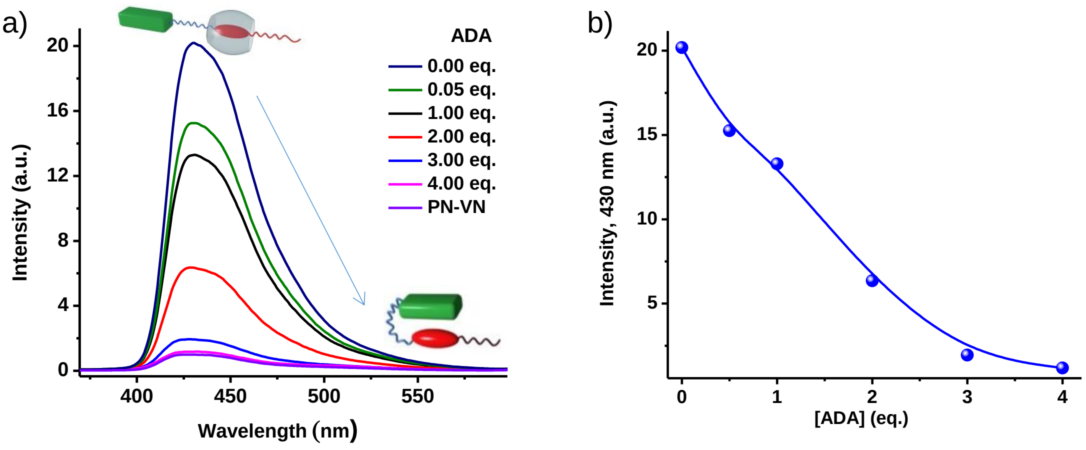

Figure S16. a) Emission spectra of **PN-VN** upon addition of Adamantyl amine (ADA) to the mixture of (**PN-VN** + 4 equiv. of CB[7]). b) Plot of emission intensity at 430 nm vs. equivalent addition of ADA ([**PN-VN**] = 10^{-4} M).

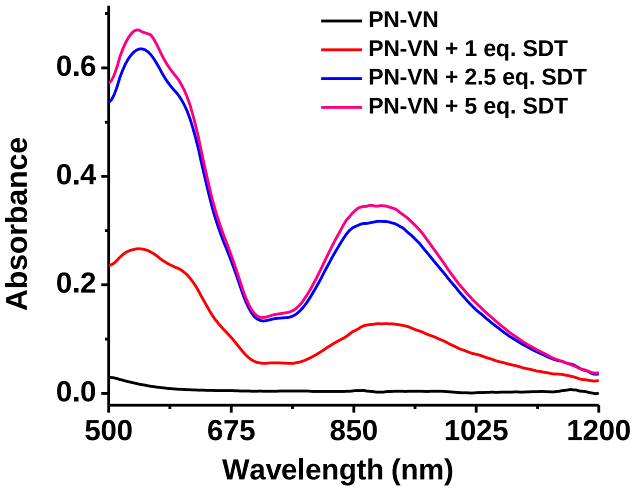

Figure S17. a) Absorption spectra depicting the formation of radical cation assembly of **PN-VN** (λ = 900 nm) with the equivalent addition of **SDT** ([**PN-VN**] = 10-4 M, H_2O, pH = 7 aqueous buffer).

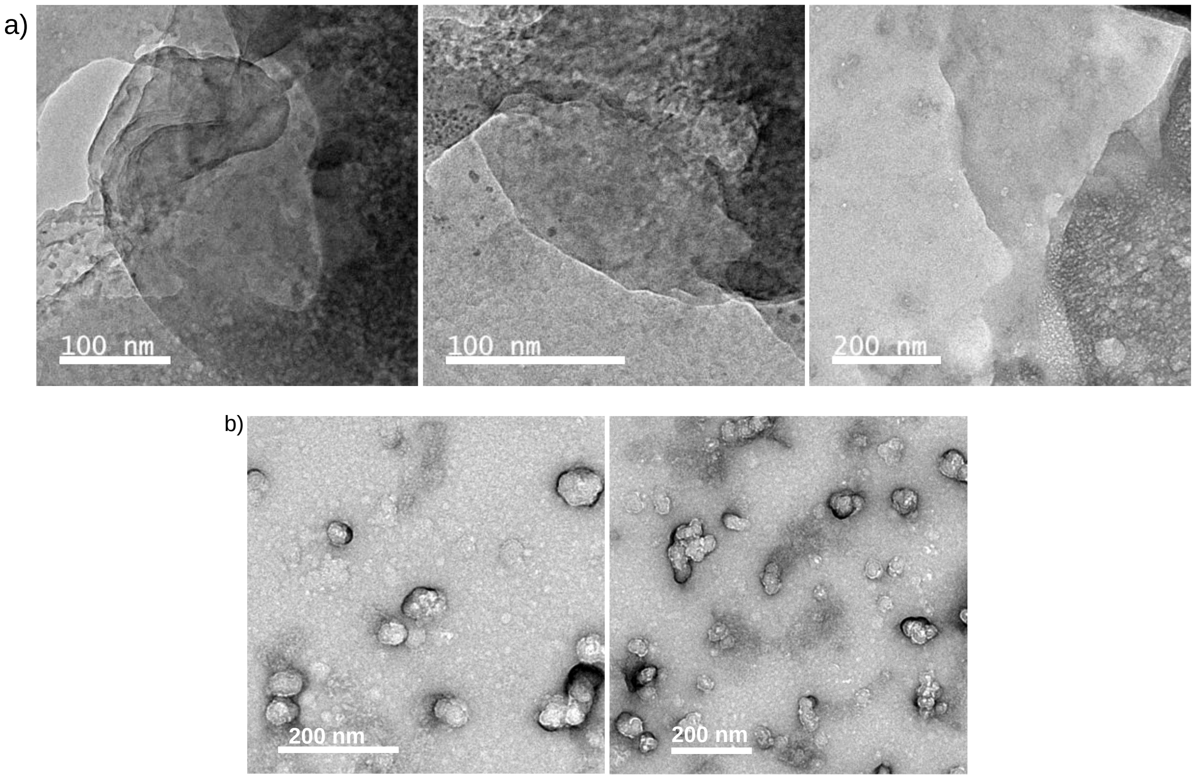

Figure S18. TEM images of a) sheet obtained after reduction of **PN-VN** with 20 eq. of **SDT**, b) vesicle obtained after reduction of **PN-VN** with 10 eq. of **SDT** and non-enzymatic oxidation by dissolved oxygen (pH = 11.5 , [**PN-VN**] = 10^{-4} M).

Note: pH 11.5 and 20 eq. of **SDT** was used for higher stability of $VN^{•+}$ to trap the transient sheet state for microscopy measurements. The quality of the sheet or vesicles has gone down because of the presence of the excess **SDT** salt in the solution.

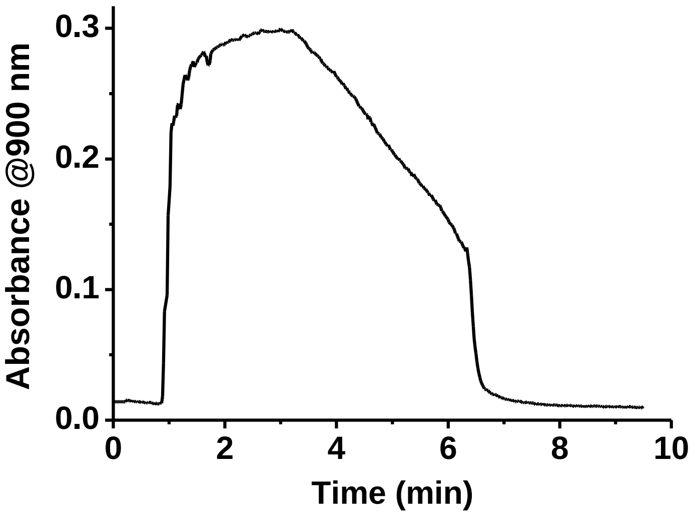

Figure S19. Absorption changes at λ = 900 nm (characteristic of $VN^{•+}$ assembly) depicting transient conformational switching in presence of 3 eq. of **SDT** and dissolved O_2 in a closed cuvette.

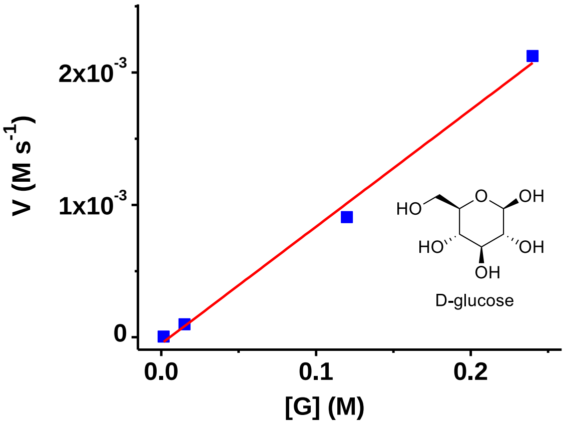

Figure S20. Michaelis Menten plot of GOx depicting effect of glucose as substrate on rate of oxidation ([**PN-VN**] = 10^{-4} M, [**SDT**] = 5 eq., GOx = 100 U/mL, T = 35 °C).

Table S1. Table for the rate constants and life time

GOx<br>U/mL	G<br>eq.	SDT<br>eq.	k [a]<br>s^{-1}	τ_1[b]<br>s	τ_2[c]<br>s
-	-	3	-	297	541
-	-	5	-	424	691
-	-	7.5	-	610	885
-	-	10	-	892	1220
20	100	5	4.7×10^{-3}	381	651
100	100	5	5.8×10^{-3}	301	501
180	100	5	6.7×10^{-3}	212	370
200	100	5	6.8×10^{-3}	127	261
100	15	5	3.5×10^{-3}	180	309
100	150	5	6.5×10^{-3}	234	381
100	1200	5	7.5×10^{-3}	279	480
100	2400	5	8.8×10^{-3}	369	686

[a] k = rate constant of the final decay. [b] τ_1 = Initial decay life time. [c] τ_2 = total life time for the decay of the radical cation state of the foldamer

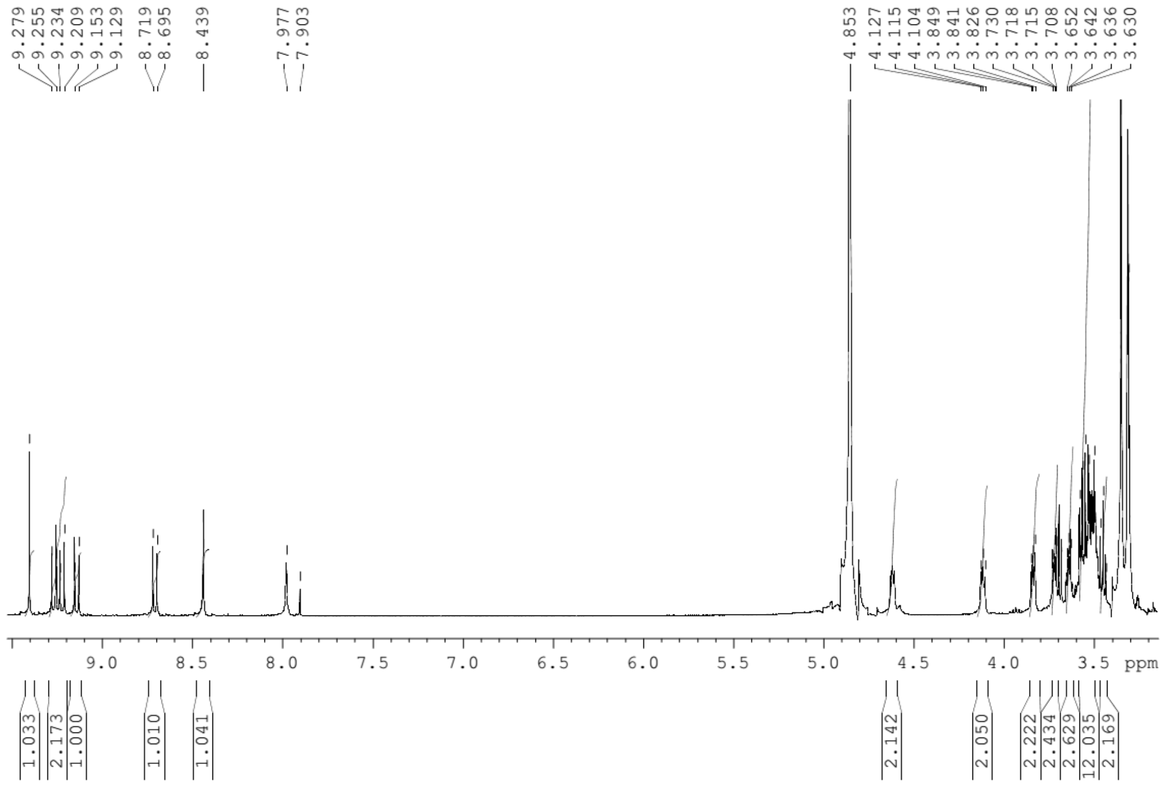

Figure S21. ^{1}H NMR of Compound **2** in deuterated MeOH.

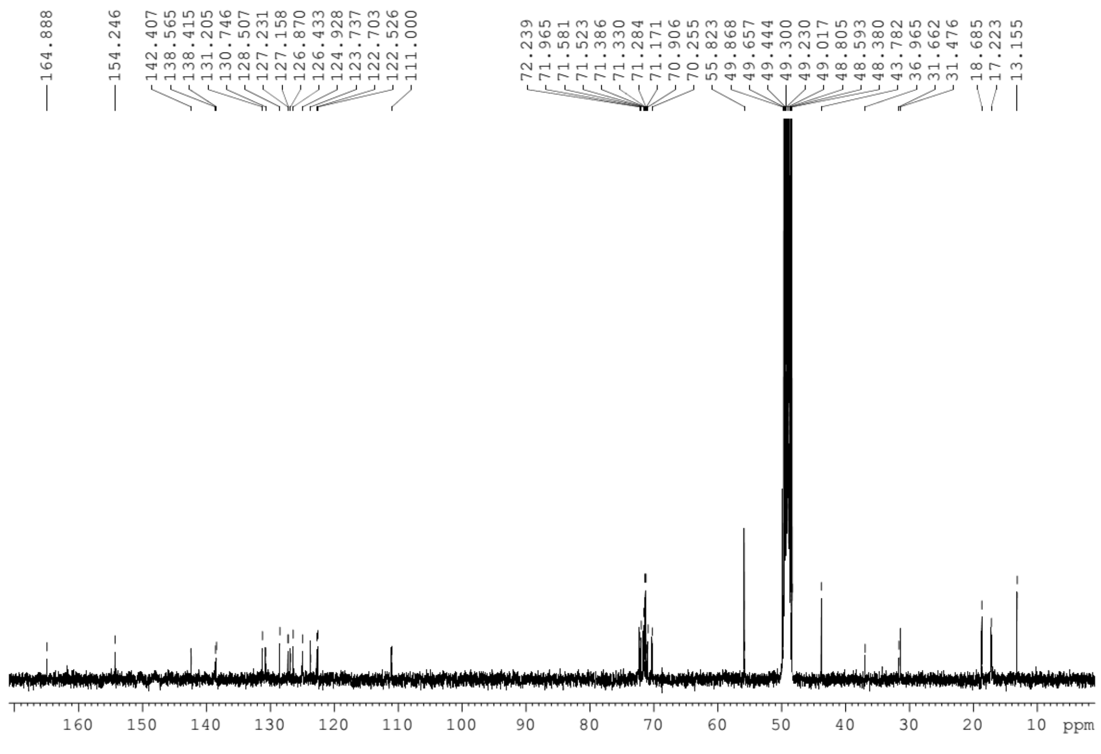

Figure S22. ^{13}C NMR of Compound **2** in deuterated MeOH.

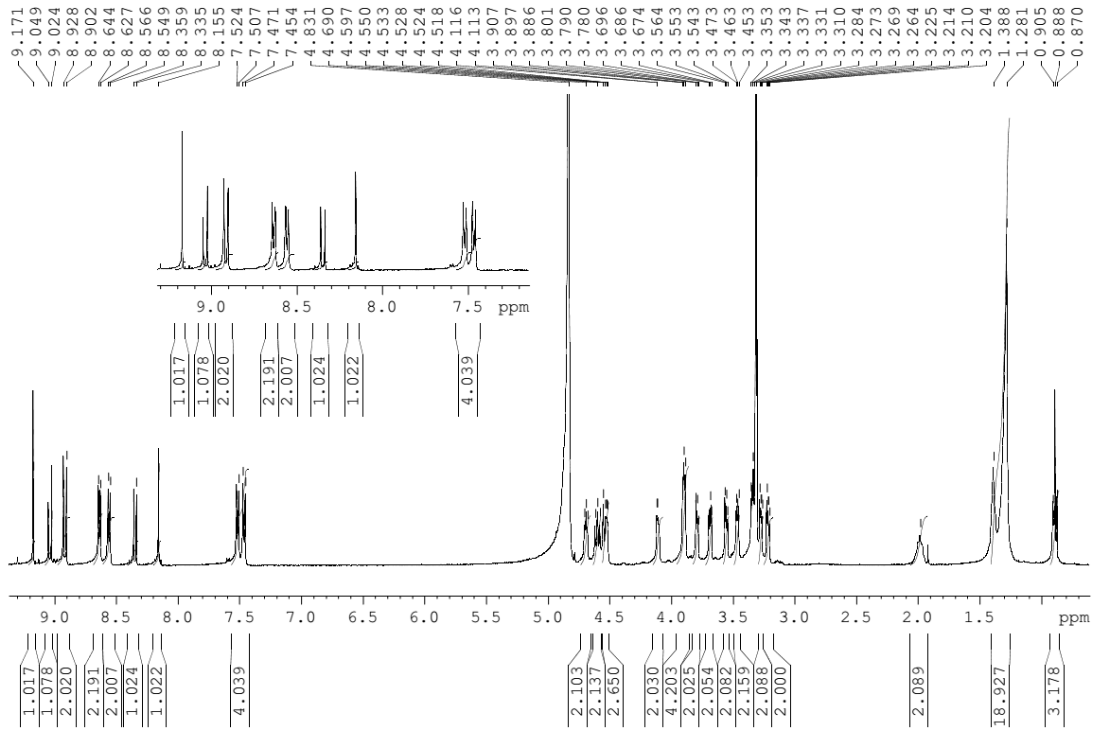

Figure S23. ^{1}H NMR of Compound **PN-VN** in deuterated MeOH

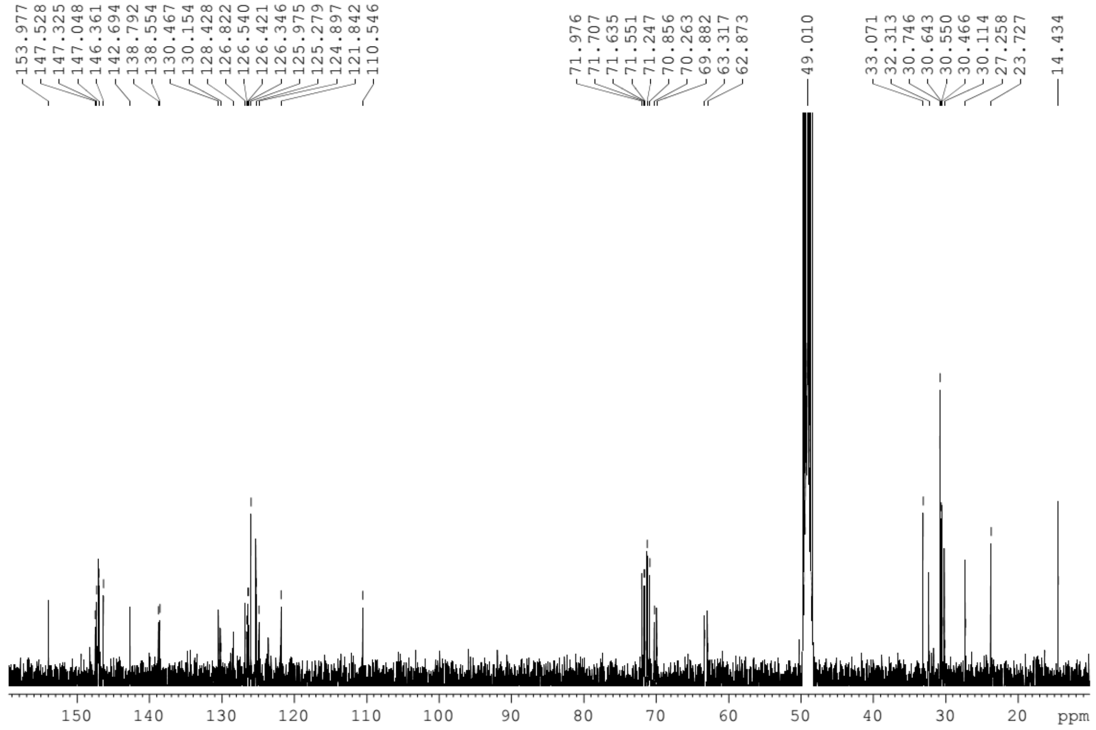

.

Figure S24. ^{13}C NMR of Compound **PN-VN** in deuterated MeOH.

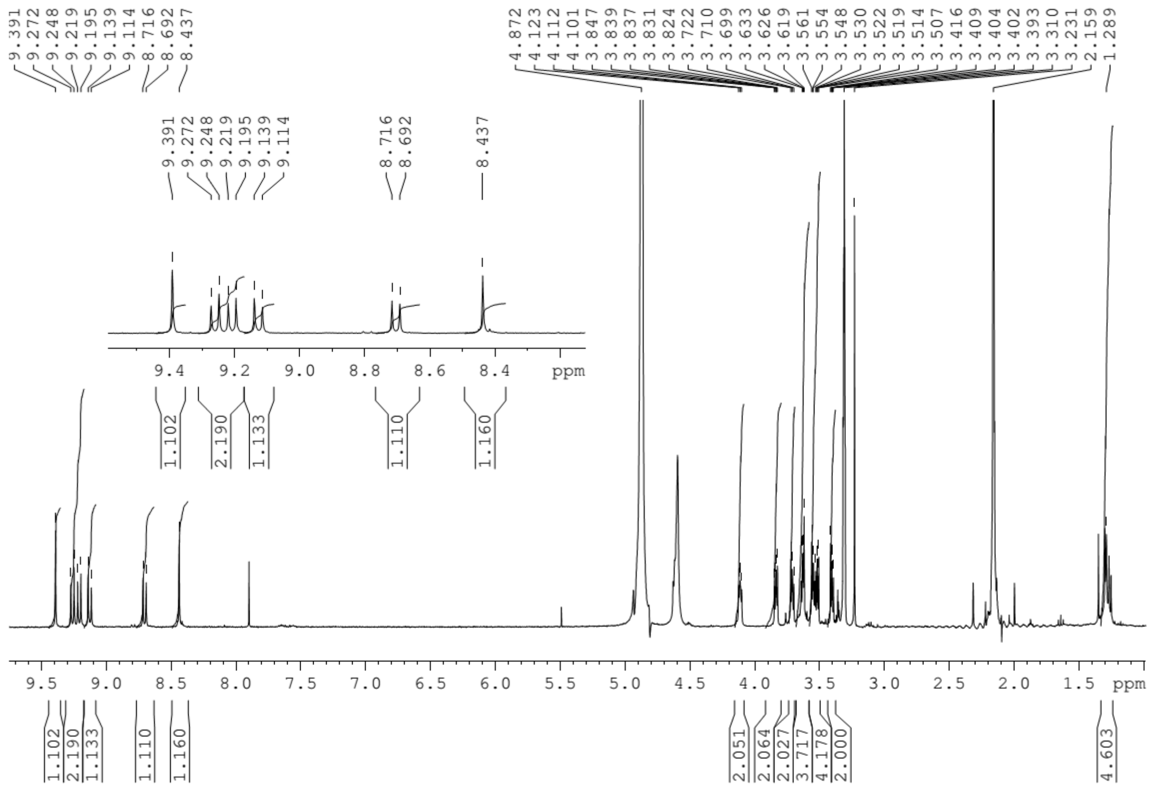

Figure S25. ^{1}H NMR spectrum of compound **PN-TEG** in deuterated MeOH

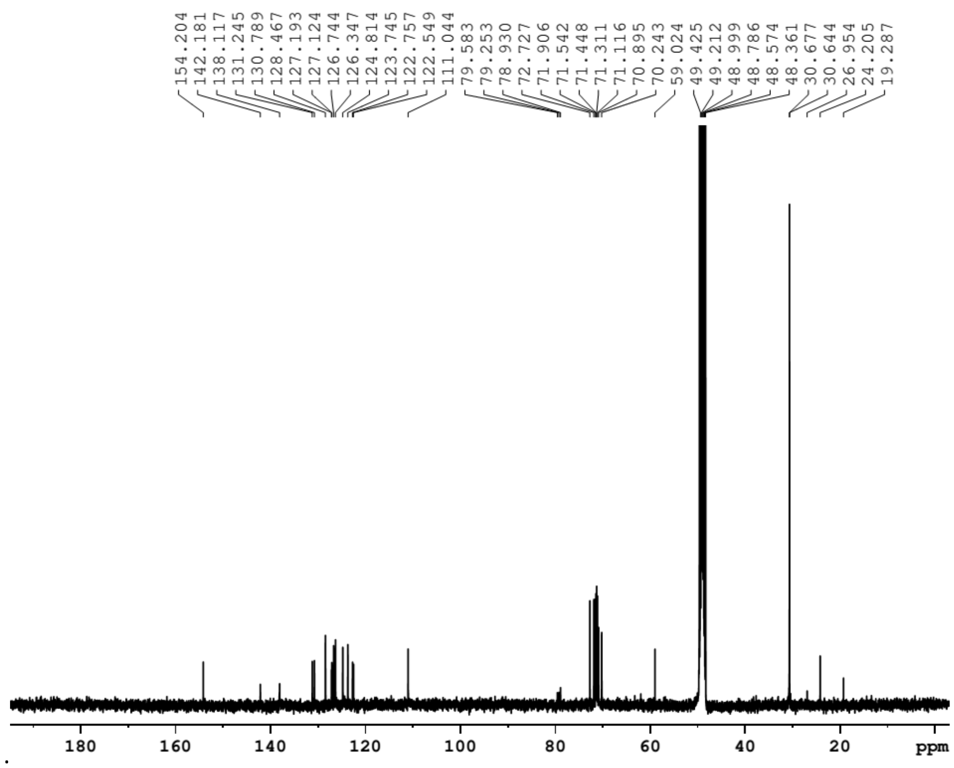

Figure S26. ^{13}C NMR of Compound **PN-TEG** in deuterated MeOH.

6. References

S1. a) C. A. Hurley, J. B. Wong, J. Ho, M. Writer, S. A. Irvine, M. J. Lawrence, S. L. Hart, A. B. Tabor and H. C. Hailes, *Org. Biomol. Chem.*, 2008, **6**, 2554–2559. b) M. F. Pepitone, G. G. Jernigan, J. S. Melinger and O. K. Kim, *Org. Lett*., 2007, **9**, 801-804.

S2. J. R. Kramer and T. J. Deming, *J. Am. Chem. Soc.*, 2014, **136**, 5547–5550.

S3. J. N. Israelachvili, *Intermolecular and surface forces*, 3rd ed.; Academic Press: London, **1992**, 535-575.

S4. a) J. Mitchel and B. W. Ninham, *J. Chem. Soc. Faraday Trans. 2,* 1981, **77**, 601-629; b) M. Antonietti and S. Forster, *Adv. Mater.*, 2003, **15**, 1323-1333. c) X. Zhang, Z. Chen and F. Würthner, *J. Am. Chem. Soc.*, 2007, **129**, 4886-4887. e) M. Ramanathan, L. K. Shrestha,T. Mori, Q. Ji, J. P. Hill and K. Ariga, *Phys. Chem. Chem. Phys*., 2013, **15**, 10580-10611.

S5. C. Tanford, *J. Phys. Chem.*, 1972, **76**, 3020−3024.

S6. N. Willis-Fox, C. Belger, J.F. Fennell Jr, R.C. Evans and T.M. Swager, *Chem. Mater.*, 2016, **28**, 2685–2691.

S7. W. J. Guan, W. J. Zhou, C. Lu and B. Z. Tang, *Angew. Chem. Int. Ed.*, 2015, ***54***, 15160–15164.

S8. a) P. Thordarson, *Chem. Soc. Rev.*, 2011, **40**, 1305-1323. b) *P. Thordarson, in Supramolecular Chemistry: From Molecules to Nanomaterials*, ed. P. Gale and J. Steed, John Wiley and Sons, Germany, 2012, vol. 8, ch. 2, pp. 239-274.

S9. K. V. Rao, K. Jayaramulu, T. K. Maji, S. J. George, *Angew. Chem. Int. Ed*., 2010, **49**, 4218–4222.

S10. C. Wang, Y. Guo, Y. Wang, H. Xu, R. Wang and X. Zhang, *Angew. Chem. Int. Ed.*, 2009, **48**, 8962-8965.
